# Genome-Wide Association Studies of Growth and Carcass Traits in Charolais Cattle Based on High-Coverage Whole-Genome Resequencing

**DOI:** 10.3390/ijms262311411

**Published:** 2025-11-25

**Authors:** Feng Zhang, Chengmei Wang, Aishao Shangguan, Xiaojun Suo, Mengjie Chen, Hu Tao, Fan Jiang, Tian Xu, Nian Zhang, Zaidong Hua, Jin Chai, Qi Xiong

**Affiliations:** 1 Hubei Key Laboratory of Animal Embryo Engineering and Molecular Breeding, Institute of Animal Husbandry and Veterinary, Hubei Academy of Agricultural Sciences, Wuhan 430064, China; zhangfeng0130@163.com (F.Z.); aishao_shangguan@163.com (A.S.); suoxj@126.com (X.S.); mengjiechen2020@163.com (M.C.); taohu@hbaas.com (H.T.); fjiang_hzau@163.com (F.J.); xutian@hbaas.ac.cn (T.X.); zn991830@163.com (N.Z.); zaidonghua@163.com (Z.H.); 2 Key Laboratory of Swine Genetics and Breeding of the Agricultural Ministry, Key Laboratory of Agricultural Animal Genetics, College of Animal Science and Technology, Huazhong Agricultural University, Wuhan 430070, China; lwchengmei@163.com

**Keywords:** growth traits, carcass traits, whole-genome resequencing, GWAS, SNP, candidate genes, Charolais cattle

## Abstract

Growth and carcass traits are key economic traits in beef cattle production, and identifying their associated genetic markers is crucial for improving breeding efficiency. Charolais cattle, as a superior beef breed, exhibit excellent performance in growth rate and meat production. The aim of this study was to utilize the preferred high-coverage whole-genome resequencing (hcWGS) as a replacement for single nucleotide polymorphism (SNP) chips to identify significant SNPs and candidate genes associated with growth (body weight, body height, cross height, body length, and chest measurement across different growth stages) and carcass traits (live backfat thickness and eye muscle area at 18 months) in 240 Charolais cattle, thereby providing guidance for beef cattle breeding. Through hcWGS (approximately 13× coverage) and quality control, 4,088,633 SNPs were identified and subsequently used for genetic analyses. Through FarmCPU-based genome-wide association studies, 196 potentially significant SNPs associated with growth traits and 29 SNPs with carcass traits were identified. Annotation analyses revealed 353 candidate genes (such as RBM33, KCTD17, PTHLH, RAC2, CHD6, TRDN, WBP1L, TLL2, CH25H, and ST13) linked to growth traits and 26 candidate genes linked to carcass traits (such as CHST11, LRRK2, RIOK2, and INTS10). Additionally, three SNPs (g.8674692C>G, g.54418624G>T, and g.71085551G>A) were validated via polymerase chain reaction–restriction fragment length polymorphism (PCR-RFLP), enabling efficient marker-assisted selection. Furthermore, eight SNPs in the Acyl-CoA oxidase 1 (ACOX1) gene were found to be associated with growth and backfat thickness traits. These findings provide valuable preliminary insights into the genetic mechanisms underlying growth and carcass traits in Charolais cattle, facilitating genome-assisted breeding.

## 1. Introduction

Cattle are important livestock species that have provided mankind with meat, milk, skin, labor, etc. However, due to the long reproductive cycle and low reproductive rates of beef cattle, breeding progress remains slow. By leveraging molecular breeding techniques to enable early selection of cattle, the breeding process can be effectively accelerated. Growth and carcass traits are very important in beef cattle breeding and considerably affect resource efficiency and farmers’ profits. Moreover, bovine growth and carcass traits generally exhibit moderate to high heritability, making the selection of specialized breeds within breeding programs a crucial strategy for enhancing these traits in cattle populations. Charolais cattle are widely recognized as one of the most prominent large-sized beef cattle breeds globally, and they have also become one of the most popular and geographically widespread cattle breeds worldwide. They are characterized by rapid growth, high meat yield, high dressing percentage, substantial body size, and strong adaptability. Charolais beef is distinguished by its high protein content and exceptional nutritional value, making it a preferred choice for germplasm improvement programs in numerous countries worldwide [1,2,3,4].

Genome-wide association studies (GWASs) are an effective and powerful genetic tool for identifying genetic markers associated with traits of interest. Numerous GWASs have been used to investigate growth or/and carcass traits in various beef cattle breeds such as Simmental [5], Chinese Simmental [6], Piemontese [7], U.S. Red Angus [8], Sussex cattle [9], Hawai’i beef cattle [10], Montana Tropical Composite beef cattle [11], Canchim beef cattle [3,12], crossbred beef cattle [13], and multiple beef cattle breeds [14,15,16]. In Charolais cattle, comparatively few studies about growth or carcass traits have been reported. The growth traits of Charolais cattle in Mexico, including birth weight, weaning weight, yearling weight, preweaning average daily gain, and postweaning average daily gain, were investigated using a 77 K single nucleotide polymorphism (SNP) chip [4]. The important traits of Irish Charolais and Limousin cattle, including carcass weight, carcass conformation, carcass fat, cull-cow weight, live weight, and feed intake, were analyzed by the custom-built International Dairy and Beef SNP chip [2]. The feed efficiency and slaughter traits in Charolais cows of France were investigated by the BovineSNP50 SNP chip [17].

With ongoing advances in high-throughput sequencing technologies and declining costs, whole-genome sequencing (WGS) is becoming the preferred method for studying livestock population evolution and molecular genetic breeding [18,19,20]. WGS is used to sequence the genomes of different individuals of a species with known gene sequences, and further analyze the variation information in the genomes of individuals by comparing the sequenced sequences with the reference genomes of the species to obtain variability information. Compared to SNP chips, WGS captures most genomic variation and eliminates the need for sequential genetic testing, with higher sensitivity and accuracy of detection. Although GWASs have explored growth or carcass traits in Charolais cattle [2,4,17], these studies were exclusively based on SNP chip genotyping and did not fully cover relevant phenotypic traits. Therefore, it is necessary to perform a more comprehensive analysis of additional production traits in Charolais using WGS approaches.

This study aims to apply the WGS method to screen and identify significant SNPs and candidate genes associated with both growth traits at multiple growth stages and carcass traits, so as to guide beef cattle breeding. First, high-coverage whole-genome sequencing (hcWGS, ~13×) was performed to obtain high-resolution genome-wide genotypes from 240 purebred Charolais cattle. Furthermore, a GWAS was conducted to identify genomic regions influencing growth and carcass traits in this population. Growth traits included body weight (BW), body height (BH), cross height (CH), body length (BL), and chest measurement (CM), which were measured in newborns (NBs) as well as at 3, 6, 12, and 18 months of age. Carcass traits comprised live backfat thickness (BFT) and eye muscle area (EM) at 18 months. Meanwhile, the annotated candidate genes were analyzed by Gene Ontology (GO) and Kyoto Encyclopedia of Genes and Genomes (KEGG) enrichment. Subsequently, three representative SNP variants were selected for effect analysis, and a polymerase chain reaction–restriction fragment length polymorphism (PCR-RFLP)-based genotyping method was developed. Finally, the association between Acyl-CoA oxidase 1 (ACOX1) gene polymorphism and growth traits in Charolais cattle was analyzed. This study identified multiple statistically significant SNPs and candidate genes related to the aforementioned traits in Chinese Charolais cattle. These findings provide a reference for understanding genetic mechanisms and improving the breeding of growth and carcass traits, while also establishing an important foundation for developing genomic selection technologies of Charolais.

## 2. Results

### 2.1. Phenotypic Statistics Analysis

The phenotypic values of BW, BH, CH, BL, and CM measured in newborns and at 3, 6, 12, and 18 months of age, as well as BFT and EM measured at 18 months, are shown in Table 1. The frequency distribution histograms of the phenotypic values for each individual trait are presented in Supplementary Figures S1–S6. These results indicated that all traits followed an approximately normal distribution and were thus suitable for subsequent GWASs.

Meanwhile, correlation analyses were conducted to elucidate the relationships among phenotypic traits (BW, BH, CH, BL, and CM). The results revealed that there were significant positive correlations among all measured traits at each age interval (*p* < 0.01) (Supplementary Tables S1–S5). Notably, the correlation between BW and CM was the strongest at all developmental stages. The correlation between BH and CH was the strongest except for 6 months of age. In addition, BL had the weakest correlation with other traits.

### 2.2. WGS, SNPs Distribution and Principal Component Analysis (PCA)

All 240 samples were subjected to high-depth sequencing and a total of 8711.60 gigabases (Gb) of raw data was obtained after WGS. The average Q20 value, Q30 value, and GC content were 97.80%, 92.69%, and 43.13%, respectively. The average actual depth was 13.06×, which met our expectations. After generating WGS data, quality control procedures were performed, and 8505.66 Gb of filtered clean data were obtained. Clean reads were aligned to Bos taurus reference assembly ARS-UCD1.2; the average mapping rate, mismatch rate, and genome coverage were 99.56%, 0.39%, and 98.33%, respectively. These results demonstrate that the sequencing data exhibit high accuracy and were therefore suitable for subsequent analyses.

After a series of quality control procedures, including filtering and imputation, a total of 4,088,633 high-quality SNPs were retained, spanning chromosomes 1 to 29 (Supplementary Figure S7A). PCA was employed to assess the population structure of Charolais cattle. The analysis revealed moderate population stratification, with the first two principal components (PC1 and PC2) accounting for 11.89% and 10.31% of the total genetic variance, respectively. The relatively continuous distribution of samples in the PCA plot indicated limited population substructure, suggesting the feasibility of subsequent association analyses (Supplementary Figure S7B).

### 2.3. Genome-Wide Association Study and Candidate Gene Identification

**Figure 1:**
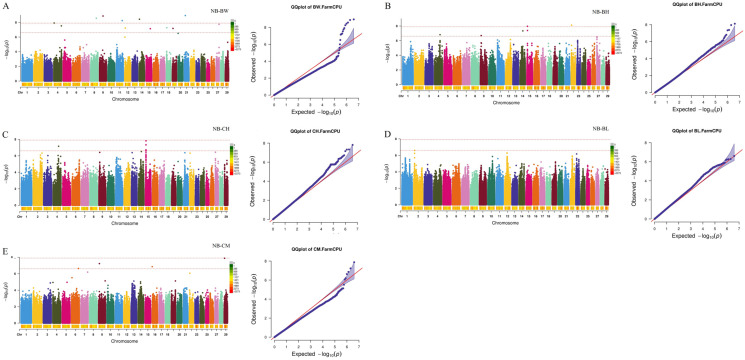
Manhattan and Q-Q plots for growth traits of newborn. (**A**–**E**) Manhattan and Q-Q plots for NB-BW (body weight of newborn), NB-BH (body height of newborn), NB-CH (cross height of newborn), NB-BL (body length of newborn), NB-CM (chest measurement of newborn), respectively. The two red dashed lines represent genome-level significance (up) and chromosomal significance (down).

**Figure 2:**
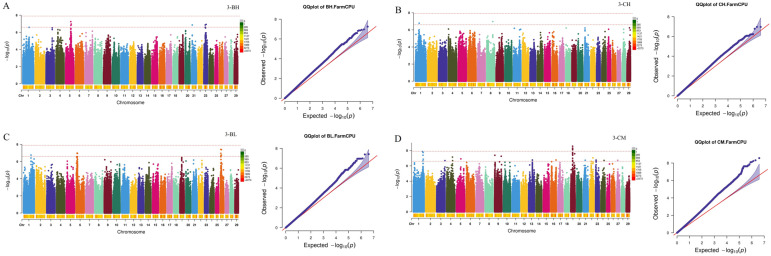
Manhattan and Q-Q plots for growth traits at the age of 3 months. (**A**–**D**) Manhattan and Q-Q plots for 3-BH (body height at the age of 3 months), 3-CH (cross height at the age of 3 months), 3-BL (body length at the age of 3 months); 3-CM (chest measurement at the age of 3 months), respectively. The two red dashed lines represent genome-level significance (up) and chromosomal significance (down).

**Figure 3:**
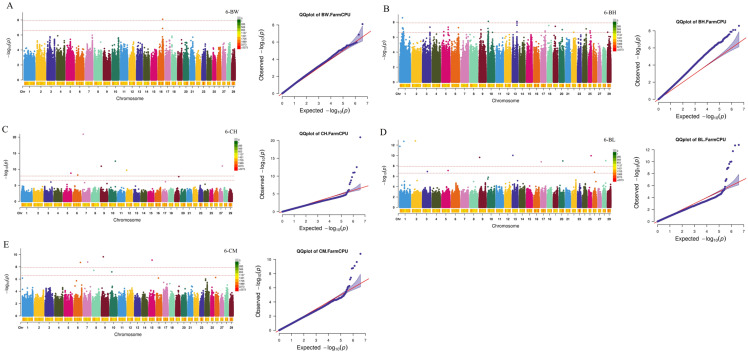
Manhattan and Q-Q plots for growth traits at the age of 6 months. (**A**–**E**) Manhattan and Q-Q plots for 6-BW (body weight at the age of 6 months), 6-BH (body height at the age of 6 months), 6-CH (cross height at the age of 6 months), 6-BL (body length at the age of 6 months), 6-CM (chest measurement at the age of 6 months), respectively. The two red dashed lines represent genome-level significance (up) and chromosomal significance (down).

**Figure 4:**
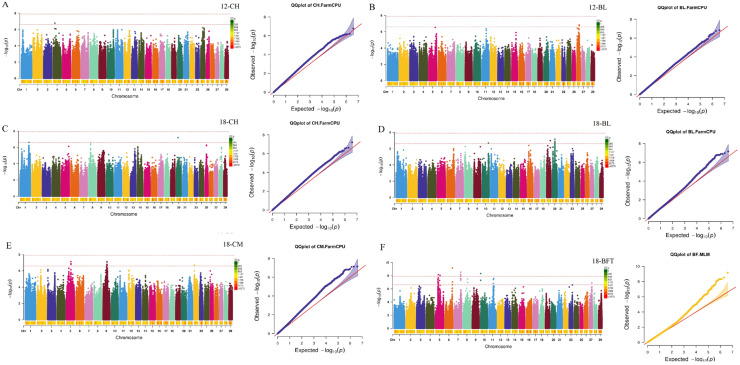
Manhattan and Q-Q plots for growth and back fat thickness traits at the age of 12 and 18 months. (**A**–**F**) Manhattan and Q-Q plots for 12-CH (cross height at the age of 12 months), 12-BL (body length at the age of 12 months), 18-CH (cross height at the age of 18 months), 18-BL (body length at the age of 18 months), 18-CM (chest measurement at the age of 18 months), 18-BFT (back fat thickness at the age of 18 months), respectively. The two red dashed lines represent genome-level significance (up) and chromosomal significance (down).

Subsequently, a GWAS for growth and carcass traits was conducted using the FarmCPU model implemented in rMVP. By setting the threshold at a *p* value of 2.44 × 10^−7^, 196 significant SNPs were identified for NB-BW, NB-BH, NB-CH, NB-BL, NB-CM, 3-BH, 3-CH, 3-BL, 3-CM, 6-BW, 6-BH, 6-CH, 6-BL, 6-CM, 12-CH, 12-BL, 18-CH, 18-BL, and 18-CM, and 29 significant SNPs were identified for 18-BFT (Figure 1, Figure 2, Figure 3 and Figure 4). Specifically, for NB-BW, NB-BH, NB-CH, NB-BL, and NB-CM, 12, 6, 1, 6, and 4 significant SNPs were identified, respectively; for 3-BH, 3-CH, 3-BL, and 3-CM, 7, 11, 3, and 42 significant SNPs were identified, respectively; for 6-BW, 6-BH, 6-CH, 6-BL, and 6-CM, 1, 44, 12, 8, and 7 significant SNPs were identified, respectively; and 2, 1, 12, 3, 14, and 29 significant SNPs were identified for 12-CH, 12-BL, 18-CH, 18-BL, 18-CM, and 18-BFT, respectively. Additionally, candidate genes within 0.1 Mb upstream and downstream of the identified SNPs were scanned. A total of 353 genes (such as RBM33, KCTD17, PTHLH, RAC2, CHD6, TRDN, WBP1L, TLL2, CH25H, and ST13) were identified to be associated with growth traits, along with 26 genes (such as CHST11, LRRK2, RIOK2, and INTS10) tied to 18-BFT (Supplementary Table S6).

### 2.4. GO and KEGG Enrichment Analyses of Candidate Genes

To systematically investigate the functional characteristics of growth- and carcass-associated candidate genes, GO annotation and KEGG pathway enrichment analyses were conducted. This integrative approach enabled the identification of significantly enriched biological processes, molecular functions, cellular components, and key metabolic pathways associated with the target gene set. A total of 137, 55, and 26 candidate genes were identified for 3-CM, 6-BH, and 18-BFT, respectively. Due to the limited number of candidate genes detected for other traits, subsequent GO and KEGG enrichment analyses were performed exclusively on these three trait-associated gene sets.

**Figure 5:**
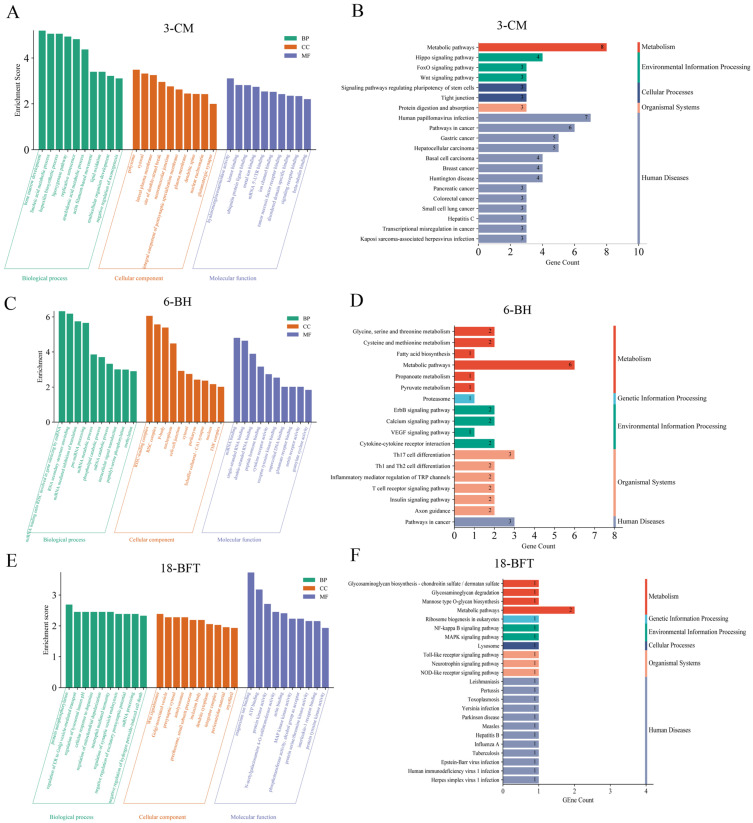
GO and KEGG enrichment analyses of candidate genes. GO (**A**) and KEGG (**B**) enrichment results for candidate genes associated with 3-CM (chest measurement at the age of 3 months); GO (**C**) and KEGG (**D**) enrichment results for candidate genes associated with 6-BH (body height at the age of 6 months); GO (**E**) and KEGG (**F**) enrichment results for candidate genes associated with 18-BFT (back fat thickness at the age of 18 months).

GO enrichment analysis was conducted for the 137 candidate genes related to 3-CM (Figure 5A). At the Biological Process level, candidate genes were mainly enriched in bone marrow development, linoleic acid metabolic process, hepoxilin biosynthetic process, etc. At the Cellular Component level, candidate genes were mainly enriched in polysome, cytosol, lateral plasma membrane, etc. At the Molecular Function level, the enrichment primarily focused on hyalurononglucosaminidase activity, kinase binding, ubiquitin protein ligase binding, etc. KEGG enrichment analysis revealed that these candidate genes were predominantly enriched in the Hippo signaling pathway, glycosaminoglycan degradation, and cardiac muscle contraction pathways (Figure 5B).

GO enrichment analysis was performed on the 55 candidate genes identified for the 6-BH trait (Figure 5C). At the Biological Process level, candidate genes were mainly enriched in miRNA loading onto RISC involved in gene silencing by miRNA, RNA secondary structure unwinding, miRNA mediated inhibition of translation, etc. At the Cellular Component level, candidate genes were mainly enriched in RISC-loading complex, RISC complex, P-body, etc. At the Molecular Function level, the enrichment primarily focused on miRNA binding, single-stranded RNA binding, double-stranded RNA binding, etc. KEGG enrichment analysis demonstrated that these candidate genes were predominantly enriched in signaling pathways, such as the VEGF signaling pathway, Insulin signaling pathway, and Glycine, Serine, and Threonine metabolism pathway (Figure 5D).

GO enrichment analysis was performed on the 26 candidate genes identified for the 18-BFT trait (Figure 5E). At the Biological Process level, candidate genes were mainly enriched in protein autophosphory, regulation of lysosomal lumen pH, etc. At the Cellular Component level, candidate genes were mainly enriched in Wnt signalosome, Golgi-associated vesicle, etc. At the Molecular Function level, the enrichment primarily focused on magnesium ion binding, ATP binding, protein kinase activity, etc. KEGG enrichment analysis indicated that these candidate genes were predominantly enriched in pathways such as the neurotrophin signaling pathway and MAPK signaling pathway (Figure 5F).

### 2.5. Functional SNPs Identification and PCR-RFLP Assays

**Figure 6:**
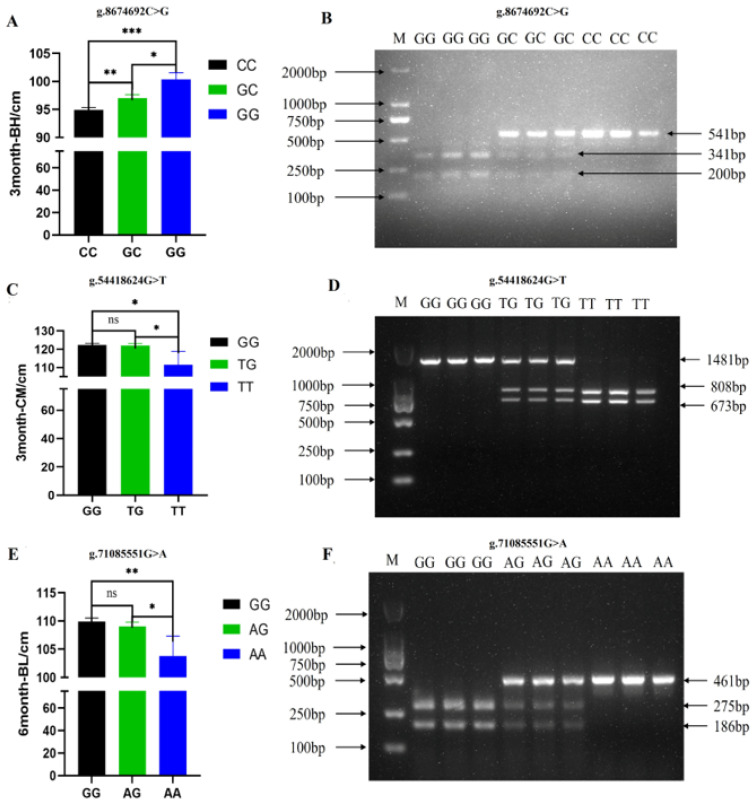
The results of SNP effect analysis and enzyme digestion typing. The effect analysis (**A**) and enzyme digestion typing (**B**) results for g.8674692C>G associated with 3-BH (body height at the age of 3 months); the effect analysis (**C**) and enzyme digestion typing (**D**) results for g.54418624G>T associated with 3-CM (chest measurement at the age of 3 months); the effect analysis (**E**) and enzyme digestion typing (**F**) results for g.71085551G>A associated with 6-BL (body length at the age of 6 months). * *p* < 0.05; ** *p* < 0.01; *** *p* < 0.001; ns, not significant.

In livestock production, genomic sequencing remains time-consuming and cost-prohibitive for routine implementation. To address this limitation, we developed a PCR-RFLP-based genotyping strategy to facilitate efficient marker-assisted selection in cattle breeding programs. This study identified three functionally significant SNPs, g.8674692C>G (associated with 3-BH), g.54418624G>T (associated with 3-CM), and g.71085551G>A (associated with 6-BL), each demonstrating clear genotype–phenotype relationships. At the g.8674692C>G locus, the 3-BH levels of the GG and GC genotypes were significantly higher than those of the CC genotype, and the GG genotype was significantly higher than the GC genotype, indicating that the GG genotype was the favorable allelic genotype (Figure 6A). Furthermore, the locus before and after mutation could be recognized by the restriction endonuclease *Hha* I. Following gel electrophoresis, three distinct band patterns were observed: the GG genotype displayed two bands of 341 bp and 200 bp, the GC genotype displayed three bands of 541 bp, 341 bp, and 200 bp, and the CC genotype displayed only one band of 541 bp (Figure 6B). Therefore, individuals with the GG genotype could be rapidly screened by the PCR-RFLP method. Similarly, at the g.54418624G>T locus, 3-CM levels were significantly higher in GG and TG genotypes than in TT, with no significant difference between GG and TG, indicating that the TT genotype was the unfavorable allele (Figure 6C). Furthermore, this locus could be cleaved by the restriction enzyme *Hin*c II before and after mutation. Gel electrophoresis revealed three distinct band patterns: the TT genotype displayed two bands, the TG genotype showed three bands, and the GG genotype yielded only a single band (Figure 6D). At the g.71085551G>A locus, individuals with the GG and AG genotypes exhibited significantly higher 6-BL levels compared to those with the AA genotype, with no significant difference observed between the GG and AG genotypes, suggesting that the AA genotype represents the unfavorable allele (Figure 6E). Furthermore, this locus can be digested by the restriction enzyme *Mnl* I. Gel electrophoresis analysis revealed three distinct banding patterns: the GG genotype produced two bands, the AG genotype displayed three bands, and the AA genotype yielded only a single band (Figure 6F). The established method enables precise identification of favorable alleles while facilitating culling of animals carrying disadvantageous genotypes, significantly enhancing breeding efficiency.

### 2.6. ACOX1 Gene Polymorphism Identification and Trait Association Analysis

**Figure 7:**
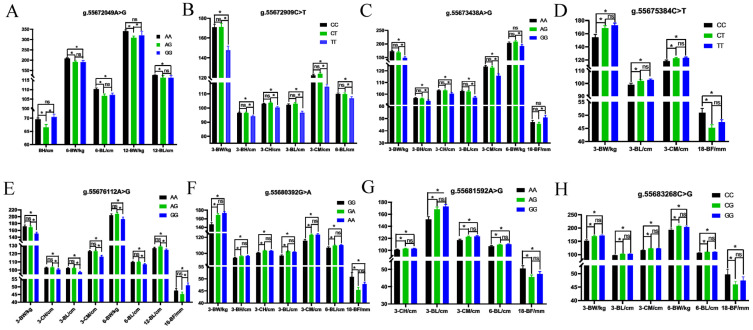
Association analysis of ACOX1 SNPs with growth and carcass traits in Charolais cattle. (**A**) g.55672049A>G; (**B**) g.55672909C>T; (**C**) g.55673438A>G; (**D**) g.55675384C>T; (**E**) g.55676112A>G; (**F**) g.55680392G>A; (**G**) g.55681592A>G; (**H**) g.55683268C>G. * *p* < 0.05; ns, not significant.

In the ACOX1 gene, eight SNPs associated with growth or carcass traits were successfully identified. Specifically, three SNPs, g.55672049A>G, g.55672909C>T, and g.55673438A>G, were localized in intron 2, and the remaining five SNPs, g.55675384C>T, g.55676112A>G, g.55680392G>A, g.55681592A>G, and g.55683268C>G, were mapped to intron 3. These identified SNPs were further analyzed to determine their associations with multiple growth and carcass traits, including NB-BW, NB-BH, NB-CH, NB-BL, NB-CM, 3-BW, 3-BH, 3-CH, 3-BL, 3-CM, 6-BW, 6-BH, 6-CH, 6-BL, 6-CM, 12-BW, 12-BH, 12-CH, 12-BL, 12-CM,18-BF, and 18-EM. The traits significantly associated with the SNPs are displayed in Figure 7A–H. These results demonstrate that ACOX1 was a promising candidate gene for genetic improvement in beef cattle, with significant associations between its polymorphisms and key growth and carcass traits. The eight identified SNPs may serve as valuable molecular markers for marker-assisted selection (MAS) in the Charolais cattle breeding program. Incorporating these variants into genomic selection strategies may enhance the efficiency of breeding for superior production traits.

## 3. Discussion

The Charolais cattle breed, renowned for its large body size and high meat yield, serves as an ideal model for investigating genetic mechanisms underlying economically important traits in beef production. Growth and carcass traits are pivotal determinants of economic efficiency in beef production systems. The identification of genetic markers, particularly SNPs and their associated genes, provides valuable tools for marker-assisted selection programs. Such genomic approaches enable more precise breeding strategies to improve production traits and meet growing global demands for high-quality beef. In this study, high-quality SNPs were obtained from a population of 240 Charolais cattle using the hcWGC methods. GWAS was then conducted to investigate the relationship between these SNPs and growth and carcass traits, leading to the identification of 225 associated SNPs and 379 candidate genes.

### 3.1. Candidate Genes Identified for Growth Traits According to the GWAS Results

Growth traits crucial for livestock development include body weight, body size, and daily weight gain. It is widely acknowledged in the industry that birth, 3-month-old, 6-month-old, 12-month-old, and 18-month-old stages are critical growth nodes in beef cattle. BW, BL, BH, CH, and CM serve as key indicators for evaluating beef cattle growth status, and all of which exhibit positive correlations with production performance. Using FarmCPU-based GWAS, we identified 196 significant SNPs and 353 candidate genes related to growth traits in Charolais cattle. We observed inconsistencies in the SNPs identified for the same traits at different time points, such as in 3-CM and 6-CM. One possible explanation for this is that genetic influences on chest measurement may vary between these time points owing to biological changes as the organism grows. Additionally, variations in sample size and statistical power at each time point may have contributed to the identification of different SNPs. Furthermore, changes in genetic interactions and environmental factors over time may contribute to variations in SNPs associated with the same trait at different developmental stages. Among the 353 candidate genes, RBM33 has been identified as being significantly associated with obesity in Northern Han Chinese by targeted resequencing technology [21], and as an RNA-binding protein, RBM333 regulates m^6^A methylation modification [22], an essential post-transcriptional regulator implicated in skeletal myogenesis [23]. KCTD17 was implicated in the cellular growth, adipogenic differentiation, and vertebrate development [24,25,26]. PTHLH plays an important role in chondrogenesis, brachydactyly, short stature, and developmental delay [27,28,29]. Similarly, RAC2 has been reported to regulate myeloid cell dysfunction, bone development, and metabolism [30,31]. In addition, CHD6 was screened to participate in porcine embryonic muscle development through comprehensive analysis of the transcriptome and miRNAome [32]. TRDN was detected to be significantly associated with adipogenesis [33,34], and it has been proposed as a candidate gene for the 6-month weight of sheep and meat quality traits of Simmental cattle [35,36]. The copy number of the WBP1L gene was significantly correlated with growth traits of Chinese cattle populations [37]. Other relevant genes, such as TLL2, CH25H, and ST13, affect muscular atrophy and adipocyte differentiation [38,39,40]. The identified genes may offer vital insights into selecting and breeding beef cattle with desirable growth traits. However, further validations and investigations are required to elucidate their precise functions and mechanisms of action.

### 3.2. Candidate Genes Identified for BFT According to the GWAS Results

Backfat thickness and eye muscle area serve as essential indicators, directly influencing meat quality and carcass yield [41]. The amount of BFT deposited on the carcass is related to the total body fat and plays a major role in beef’s flavor and juiciness and dressing percentage [42]. In this study, although no SNPs associated with EM were detected, 29 significant SNPs and 26 candidate genes related to BFT were identified. Important genes include CHST11, which contains two functional intronic PPARγ binding sites and exhibits reduced intracellular lipid accumulation in mature adipocytes following its knockdown [43]; LRRK2, which regulates CPT1A expression to promote β-oxidation and thereby facilitates lipid catabolism in HepG2 cells [44], and also mediates Rab8a phosphorylation to promote lipid storage in 3T3 cells [45]; RIOK2, which as an ATPase plays a pivotal role in regulating cell growth and protein synthesis across diverse cellular contexts [46,47]; and INTS10, whose causal variant rs112861901 influences high-density lipoprotein levels in bodily metabolic processes as identified through GWAS [48]. The pathways identified by GO and KEGG enrichment analyses corresponded closely to these biological processes, including regulation of synaptic vesicle endocytosis, ATP binding, protein serine/threonine kinase activity, metabolic pathways, and the MAP signaling pathway. Thus, we speculated that these genes might affect backfat thickness by regulating the lipid metabolism. The identified backfat thickness-related candidate genes may provide an opportunity to accelerate the meat quality and carcass yield of Charolais cattle.

### 3.3. PCR-RFLP for Quick Identification of SNPs

PCR-RFLP is the simplest and fastest method for single-nucleotide change detection, and it has been widely applied in livestock, including sheep [49], pigs [50], and crossbred cattle [51]. In the current study, three functionally significant SNPs, g.8674692C>G, g.54418624G>T, and g.71085551G>A, were genotyped using PCR-RFLP with restriction enzymes *Hha* I, *Hin*c II, and *Mnl* I, respectively. Based on the results of genotype–phenotype association analysis, after digestion with *Hha* I, if gel electrophoresis shows only two bands (341 bp and 200 bp), it indicates that the genotype of the g.8674692C>G locus in Charolais individuals is the advantageous genotype GG, and such individuals can be prioritized as candidate breeding cattle. Similarly, after digestion with *Hin*c II, gel electrophoresis displays one band (1481 bp) or three bands (1481 bp, 808 bp, and 673 bp); this suggests that the genotype of the g.54418624G>T locus in Charolais individuals is the advantageous genotype GG or TG, and these individuals can be given priority consideration as candidate breeding cattle. After digestion with *Mnl* I, gel electrophoresis reveals two bands (275 bp and 186 bp) or three bands (461 bp, 275 bp, and 186 bp); this implies that the genotype of the g.71085551G>A locus in Charolais individuals is the advantageous genotype GG or AG, and such individuals can be focused on as candidate breeding cattle.

### 3.4. ACOX1 Polymorphism and Association Analysis

ACOX1 is the first and rate-limiting enzyme in the peroxisomal fatty acid β-oxidation pathway across all eukaryotes [52]. Our previous studies have demonstrated that ACOX1 promotes adipogenesis in bovine preadipocytes [53]. Additionally, prior studies have reported that polymorphisms in the ACOX1 gene were associated with the backfat thickness and meat quality grade in Chinese indigenous cattle and cultivated cattle breeds, and Korean cattle. One SNP in exon 13 of the bovine ACOX1 gene resulted in significant differences in ultrasound backfat thickness and ultrasound marbling score among different genotypes [54]. The g.224G > A SNP located in ACOX1 coding regions was significantly associated with meat quantity grade at slaughter and backfat thickness tended to be greater in Korean cattle [55]. The association between ACOX1 gene polymorphism and growth traits in Charolais cattle has never been studied. In the present study, eight polymorphic loci were identified in the Charolais cattle population, all of which are located in intronic regions. However, relatively few SNPs were associated with traits at birth or 12 months of age. Specifically, only the g.55672049A>G locus showed a significant difference in body height traits at birth, and only the g.55672049A>G and g.55676112A>G loci exhibited significant differences in body weight or body length traits at 12 months of age. In contrast, all eight loci were significantly associated with one or more traits at 3 months and 6 months of age. This suggests that molecular marker loci associated with target growth traits could be selected for early selection of the Charolais cattle population at 3 months and 6 months of age, which was consistent with previous research results [56]. SNPs located in intronic regions may exert significant impacts on gene expression and phenotypes through multiple complex mechanisms. They can directly regulate gene transcriptional efficiency by creating or disrupting binding sites for transcription factors, enhancers, or silencers; induce alternative splicing and isoform imbalance by affecting pre-mRNA splice sites or regulatory elements; and interfere with the production of functional non-coding RNAs, or exert distal regulation by altering chromatin spatial conformation and epigenetic modifications. The detailed mechanisms underlying the functional roles of SNPs in ACOX1 remain to be further elucidated.

### 3.5. Application of SNPs and Candidate Genes in Future Genomic Breeding of Beef Cattle

The SNPs and candidate genes identified through hcWGS in this study provide a genetic foundation for precision breeding in beef cattle. These discoveries enable MAS for key economic traits such as growth and carcass characteristics, permitting the early identification of superior individuals to accelerate genetic gain. Furthermore, they can enhance the accuracy of genomic selection (GS) models by refining the prediction of genomic estimated breeding values (GEBVs) for complex traits. The candidate genes also present direct targets for gene editing initiatives. For broad industry application, core SNPs can be incorporated into cost-effective genotyping platforms. Collectively, these resources empower a shift from traditional phenotype-based selection to genomics-informed breeding strategies, facilitating the synergistic improvement of multiple traits and promoting sustainable genetic progress in beef cattle populations.

## 4. Materials and Methods

### 4.1. Animals and Phenotype Data

A total of 240 Charolais cattle, including 69 bulls and 171 cows Charolais cattle, were obtained from the national core breeding farm in Hubei Province, China. These cattle were provided with a uniform diet and subjected to a standardized raising and management protocol, with ad libitum feeding. The experimental procedure was approved by the Animal Care and Use Committee of the Hubei Academy of Agricultural Sciences, China, and was conducted in accordance with animal welfare and ethics guidelines (No. HBAASEC-2023-011, 16 February 2023).

The measured traits of this population were as follows: BW, BH, BL, CH, and CM at five key growth stages of newborn, 3 months, 6 months, 12 months, and 18 months, as well as BFT and EM at 18 months. All weight-related traits were measured after a 12 h fasting period, all body height-related traits were measured from the highest point of the withers to the ground, body length-related traits were measured from the front edge of the shoulder to the trailing edge of the sitting bone, cross height-related traits were measured from the highest point of the hooks to the ground, and all chest measurement-related traits were measured by encircling the trailing edge of the scapula. BH, BL, CH, and CM were measured on the same flat surface, and the cattle were maintained in a natural standing posture during the measurement. Then the phenotypic distribution statistics were analyzed to determine whether the above phenotypic data conform to a normal distribution.

### 4.2. Whole-Genome Resequencing

Cervical venous blood was drawn from the 240 Charolais cattle, and 3–5 mL of fresh blood was used to extract genomic DNA using the DNA Extraction Kit of Genstone Biotech (TD324). DNA samples with concentration ≥ 40 ng/μL, quantity ≥1 μg, A260/A280 ratio between 1.8 and 2.0, and that passed gel electrophoresis test were used for whole-genome resequencing. After assessing the DNA quality, gene libraries were assembled, each with an average fragment size of 300 bp per sample. The sequencing was carried out by the Beijing Genomics Institute using the DNBSEQ-T7 sequencer with high-depth sequencing (~13×) to obtain the raw downstream data.

### 4.3. Alignment, Variant Identification and PCA

After sequencing, Fastp (version 0.19.7) [57] was used to filter the raw data using default parameters. The clean reads obtained after quality control were aligned to Bos taurus reference assembly ARS-UCD1.2 using the BWA-MEM algorithm of the Burrows-Wheeler Aligner v0.7.17- r1188 [58]. The Picard tools (http://broadinstitute.github.io/picard, accessed on 20 January 2024) were used to filter potential duplicate reads. The “Haplotype Caller”, Genotype GVCFs”, and “Select Variants” modules of the Genome Analysis Toolkit (GATK, version 4.2.0.0) were used to call single nucleotide polymorphisms (SNPs). The filtration of raw SNPs was conducted by using the “variant Filtration” module of GATK. Finally, quality control of the SNPs was conducted using PLINK (version 1.90) software [59] with the following parameters: –geno 0.1, –mind 0.1, –maf 0.01, and –hwe 0.000001. PCA was used to evaluate cattle population stratification based on WGS data from 240 individuals using PLINK.

### 4.4. Genome-Wide Association Study

GWAS was performed using R package rMVP (version 1.0.0) using the Fixed and random model Circulating Probability Unification (FarmCPU) method [27]. All parameters were set to their default values. The FarmCPU model was evaluated iteratively using the fixed-effect model (FEM) and random-effect model (REM). The FEM can be written as

*y* = *Xb* + *Z_t_u_t_* + *S_i_d_i_* + *e*

where *y* is a vector of phenotypes of the analyzed trait; *b* is a vector of fixed effects and covariates, including top three principal components, sex, and farm effects; *u_t_* is a vector of the fixed effects for the *tth* pseudo-QTNs; *X* and *Z_t_* are the corresponding incidence matrices for *b* and *u_t_*; *S_i_* is the genotype for the *ith* candidate SNP; *d_i_* is the effect of the *ith* candidate SNP; and *e* is a vector of the residuals. The REM updated the pseudo-QTNs as follows:

*y = u + e *

where *y* is a vector of phenotypes of the analyzed trait; *u* ~ *MVN* (0, 2*Kσ_u_^2^*) with *σu*2 being the unknown genetic variance, and *K* being the kinship matrix computed by the pseudo-QTNs; and *e* is a vector of the residuals.

Bonferroni multiple tests were employed to identify the genome-wide significant (0.05/number of SNPs) and suggestive (1/number of SNPs) SNPs. Significant association between SNPs and reproductive traits was visualized in Manhattan plots and QQ-plots generated using rMVP when conducting GWAS.

### 4.5. Candidate Gene Identification and Functional Enrichment Analysis

The candidate genes were retrieved within 0.1 Mb on either side of the significant SNPs using the gene annotation information of the beef cattle reference genome version ARS-UCD1.2 in ENSEMBL (https://asia.ensembl.org/index.html, accessed on 27 January 2024), while only protein-coding genes were retained. KOBAS-i (http://bioinfo.org/kobas/, accessed on 15 February 2024) was used to perform GO and KEGG enrichment analyses, with *p* < 0.05 considered statistically significant. Based on the enrichment results, graphing was performed using Bioinformatics (https://www.bioinformatics.com.cn/, accessed on 15 February 2024) to obtain GO enrichment and KEGG enrichment bubbles and bar graphs.

### 4.6. SNP Effect Analysis and Genotyping by PCR-RFLP

Based on GWAS, candidate significant SNP loci were selected for effect analysis. The Charolais cattle population was stratified into three genotypic groups for each polymorphism: homozygous reference (AA), heterozygous (Aa), and homozygous alternative (aa). Phenotypic trait values were aggregated for each genotype group, and summary statistics (mean ± standard deviation) were calculated. The effect size of each SNP variant on the target phenotype was quantified and visualized using GraphPad Prism (version 8.0).

To develop a robust PCR-RFLP genotyping assay, we first retrieved genomic sequences encompassing 1000 bp flanking regions (upstream and downstream) of each target SNP from the NCBI Genome Database (http://www.ncbi.nlm.nih.gov/, accessed on 22 February 2024). Potential restriction enzymes were systematically screened using NEBcutter 3.0 (https://nc3.neb.com/NEBcutter/, accessed on 22 February 2024) to identify those exhibiting differential digestion patterns between wild-type and variant alleles. Enzymes were selected based on the following criteria: (1) generation of distinct fragment patterns for each genotype; (2) single recognition site within the amplicon; and (3) optimal activity under standard reaction conditions. Target SNP regions were amplified in PCR reactions, and then the amplicons were digested with the selected restriction enzyme at 37 °C for 1 h. Digested fragments were separated by electrophoresis on 1.5% agarose gels. Genotypes were determined by comparing observed fragment patterns with predicted digestion profiles.

### 4.7. ACOX1-SNP Association Study

The association between ACOX1-SNP and phenotypes was analyzed using a linear mixed model (LMM) framework implemented by a self-developed program in SAS (version 9.4). The model included genotype (I), sire (A), age (B), and sex (C) as fixed effects, with animal individual (D) as a random effect:

*Y_ikjlm_ = μ + I_i_ + A_k_ + B_j_ + C_l_ + D_m_ + E_ikjlm_*

where *Y_ikjlm_* represents the observed trait value, *μ* is the overall mean, *D_m_*∼*N* (0, *σ_D_*^2^) denotes the random effect of the *m*-th animal individual (normally distributed with variance *σ_D_*^2^), and *E_ikjlm_*∼*N* (0, *σ_E_*^2^) is the residual error.

## 5. Conclusions

The aim of this study was to investigate the significant SNPs and genes associated with five growth traits and two carcass traits in Charolais cattle. hcWGS was performed on 240 Charolais cattle with approximately 13× coverage. Using the FarmCPU-based GWAS, 196 and 29 potential SNPs were identified for growth and carcass traits, respectively, along with 353 and 26 candidate genes associated with these traits. In addition, to reduce costs and achieve rapid detection, a PCR-RFLP-based genotyping strategy for the g.8674692C>G, g.54418624G>T, and g.71085551G>A loci was established to facilitate early selection of breeding cattle. Lastly, eight SNPs associated with growth and backfat thickness traits were detected in the intronic region of the ACOX1 gene. These findings provide valuable preliminary insights into the genetic mechanisms underlying growth and carcass traits in Charolais cattle, and facilitate genome-assisted breeding, although further functional validation of these potential SNPs and candidate genes is required.

## 6. Patents

Some results reported in this manuscript have been filed for patent protection. The details are as follows:

[1] Feng Zhang, Chengmei Wang, Qi Xiong, et al., Application of an SNP molecular marker related to 3-month-old body height traits in beef cattle. China, ZL202410679360.2. Filing date: 29 May 2024; Authorization announcement date: February 7, 2025. Status: Granted.

[2] Feng Zhang, Chengmei Wang, Qi Xiong, et al., Application of an SNP molecular marker related to 6-month-old body length traits in beef cattle. China, ZL202410679460.5. Filing date: 29 May 2024; Authorization announcement date: August 29, 2025. Status: Granted.

## Figures and Tables

**Table 1:** Descriptive statistics of phenotypic values for the Charolais cattle of China.

Traits ^a^	Individuals	Mean ± SD ^b^	Maximum	Minimum	CV ^c^
NB-BW	142	39.99 ± 7.77	57.00	15.00	0.19
NB-BH	142	68.90 ± 5.49	80.00	50.00	0.08
NB-CH	142	74.46 ± 5.88	87.00	54.00	0.08
NB-BL	142	64.25 ± 6.01	78.00	50.00	0.09
NB-CM	142	73.94 ± 6.06	87.00	55.00	0.08
3-BW	139	166.92 ± 26.59	254.00	96.00	0.16
3-BH	139	96.03 ± 4.30	105.00	84.00	0.04
3-CH	139	102.71 ± 4.55	115.00	86.00	0.04
3-BL	139	101.45 ± 6.72	115.00	80.00	0.06
3-CM	139	121.81 ± 9.24	144.00	92.00	0.07
6-BW	135	203.50 ± 29.17	287.00	126.0	0.14
6-BH	135	100.71 ± 4.10	110.00	90.00	0.04
6-CH	135	108.70 ± 5.94	153.00	96.00	0.05
6-BL	135	109.34 ± 5.89	133.00	95.00	0.05
6-CM	135	131.98 ± 6.51	152.00	110.00	0.04
12-BW	115	336.92 ± 53.49	463.00	185.00	0.16
12-BH	115	114.10 ± 4.22	123.00	103.00	0.03
12-CH	115	121.89 ± 4.79	132.00	111.00	0.03
12-BL	115	127.00 ± 6.55	143.00	110.00	0.05
12-CM	115	158.13 ± 9.33	184.00	129.00	0.06
18-BW	100	457.25 ± 75.90	654.00	280.00	0.16
18-BH	100	123.27 ± 4.90	141.00	112.00	0.03
18-CH	100	132.40 ± 4.59	143.00	121.00	4.59
18-BL	100	141.53 ± 7.25	159.00	121.00	0.05
18-CM	100	177.67 ± 10.59	212.00	146.00	0.06
18-BFT	190	50.72 ± 10.91	84.90	2.70	0.21
18-EM	190	76.24 ± 10.23	106.70	50.70	0.13

^a^ Traits: NB-BW (kg), body weight of newborn; NB-BH (cm), body height of newborn; NB-CH (cm), cross height of newborn; NB-BL (cm), body length of newborn; NB-CM (cm), chest measurement of newborn; 3-BW (kg), body weight at the age of 3 months; 3-BH (cm), body height at the age of 3 months; 3-CH (cm), cross height at the age of 3 months; 3-BL (cm), body length at the age of 3 months; 3-CM (cm), chest measurement at the age of 3 months; 6-BW (kg), body weight at the age of 6 months; 6-BH (cm), body height at the age of 6 months; 6-CH (cm), cross height at the age of 6 months; 6-BL (cm), body length at the age of 6 months; 6-CM (cm), chest measurement at the age of 6 months; 12-BW (kg), body weight at the age of 12 months; 12-BH (cm), body height at the age of 12 months; 12-CH (cm), cross height at the age of 12 months; 12-BL (cm), body length at the age of 12 months; 12-CM (cm), chest measurement at the age of 12 months; 18-BW (kg), body weight at the age of 18 months; 18-BH (cm), body height at the age of 18 months; 18-CH (cm), cross height at the age of 18 months; 18-BL (cm), body length at the age of 18 months; 18-CM (cm), chest measurement at the age of 18 months; 18-BFT (mm), back fat thickness at the age of 18 months; 18-EM (cm^2^), eye muscle area at the age of 18 months. ^b^ SD, standard deviation. ^c^ CV, coefficient of variation.

## Data Availability

The original contributions presented in the study are included in the article/Supplementary Materials, further inquiries can be directed to the corresponding author.

## Supplementary Materials

The following supporting information can be downloaded at: https://www.mdpi.com/article/10.3390/ijms262311411/s1.

## Abbreviations

The following abbreviations are used in this manuscript:

GWAS	genome-wide association study
WGS	whole-genome sequencing
hcWGS	high-coverage whole-genome sequencing
NB	newborn
BW	body weight
BH	body height
CH	cross height
BL	body length
CM	chest measurement
BFT	back fat thickness
EM	eye muscle area
PCR-RFLP	polymerase chain reaction–restriction fragment length polymorphism
SNP	single nucleotide polymorphism
ACOX1	Acyl-CoA oxidase 1

## References

[1] de Rezende M.P.G., Malhado C.H.M., Biffani S., Carneiro P.L.S., Bozzi R. (2020). Genetic diversity derived from pedigree information and estimation of genetic parameters for reproductive traits of Limousine and Charolais cattle raised in Italy. Ital. J. Anim. Sci..

[2] Keogh K., Carthy T.R., McClure M.C., Waters S.M., Kenny D.A. (2021). Genome-wide association study of economically important traits in Charolais and Limousin beef cows. Animal.

[3] Santiago G.G., Siqueira F., Cardoso F.F., Regitano L.C.A., Ventura R., Sollero B.P., Souza M.D., Mokry F.B., Ferreira A.B.R., Torres R.A.A. (2017). Genomewide association study for production and meat quality traits in Canchim beef cattle. J. Anim. Sci..

[4] Jahuey-Martínez F.J., Parra-Bracamonte G.M., Sifuentes-Rincón A.M., Martínez-González J.C., Gondro C., García-Pérez C.A., López-Bustamante L.A. (2016). Genomewide association analysis of growth traits in Charolais beef cattle. J. Anim. Sci..

[5] Braz C.U., Rowan T.N., Schnabel R.D., Decker J.E. (2021). Genome-wide association analyses identify genotype-by-environment interactions of growth traits in Simmental cattle. Sci. Rep..

[6] Liu Y., Xu L., Wang Z., Xu L., Chen Y., Zhang L., Xu L., Gao X., Gao H., Zhu B. (2019). Genomic prediction and association analysis with models including dominance effects for important traits in Chinese simmental beef cattle. Animals.

[7] Pegolo S., Cecchinato A., Savoia S., Di Stasio L., Pauciullo A., Brugiapaglia A., Bittante G., Albera A. (2020). Genome-wide association and pathway analysis of carcass and meat quality traits in Piemontese young bulls. Animal.

[8] Smith J.L., Wilson M.L., Nilson S.M., Rowan T.N., Schnabel R.D., Decker J.E., Seabury C.M. (2022). Genome-wide association and genotype by environment interactions for growth traits in U.S. Red Angus cattle. BMC Genom..

[9] Bila L., Putra W.P.B., Malatji D.P., Sanarana Y.P., Tyasi T.L. (2024). Genome-wide association study (GWAS) for body weights of sussex cattle (*Bos taurus*) in South Africa. Heliyon.

[10] Adhikari M., Kantar M.B., Longman R.J., Lee C.N., Oshiro M., Caires K., He Y. (2023). Genome-wide association study for carcass weight in pasture-finished beef cattle in Hawai'i. Front. Genet..

[11] Grigoletto L., Ferraz J.B.S., Oliveira H.R., Eler J.P., Bussiman F.O., Abreu Silva B.C., Baldi F., Brito L.F. (2020). Genetic Architecture of Carcass and Meat Quality Traits in Montana Tropical^®^ Composite Beef Cattle. Front. Genet..

[12] Buzanskas M.E., Grossi D.A., Ventura R.V., Schenkel F.S., Sargolzaei M., Meirelles S.L., Mokry F.B., Higa R.H., Mudadu M.A., da Silva M.V. (2014). Genome-wide association for growth traits in Canchim beef cattle. PLoS ONE.

[13] Snelling W.M., Allan M.F., Keele J.W., Kuehn L.A., McDaneld T., Smith T.P., Sonstegard T.S., Thallman R.M., Bennett G.L. (2010). Genome-wide association study of growth in crossbred beef cattle. J. Anim. Sci..

[14] Wang Y., Zhang F., Mukiibi R., Chen L., Vinsky M., Plastow G., Basarab J., Stothard P., Li C. (2020). Genetic architecture of quantitative traits in beef cattle revealed by genome wide association studies of imputed whole genome sequence variants: II: Carcass merit traits. BMC Genom..

[15] Doyle J.L., Berry D.P., Veerkamp R.F., Carthy T.R., Walsh S.W., Evans R.D., Purfield D.C. (2020). Genomic regions associated with skeletal type traits in beef and dairy cattle are common to regions associated with carcass traits, feed intake and calving difficulty. Front. Genet..

[16] Vanvanhossou S.F.U., Scheper C., Dossa L.H., Yin T., Brügemann K., König S. (2020). A multi-breed GWAS for morphometric traits in four Beninese indigenous cattle breeds reveals loci associated with conformation, carcass and adaptive traits. BMC Genom..

[17] Martin P., Taussat S., Vinet A., Krauss D., Maupetit D., Renand G. (2019). Genetic parameters and genome-wide association study regarding feed efficiency and slaughter traits in Charolais cows. J. Anim. Sci..

[18] Song X., Yao Z., Zhang Z., Lyu S., Chen N., Qi X., Liu X., Ma W., Wang W., Lei C. (2024). Whole-genome sequencing reveals genomic diversity and selection signatures in Xia'nan cattle. BMC Genom..

[19] Harish A., Lopes Pinto F.A., Eriksson S., Johansson A.M. (2024). Genetic diversity and recent ancestry based on whole-genome sequencing of endangered Swedish cattle breeds. BMC Genom..

[20] Saleh A.A., Xue L., Zhao Y. (2023). Screening Indels from the whole genome to identify the candidates and their association with economic traits in several goat breeds. Funct. Integr. Genom..

[21] Wu Y., Wang W., Jiang W., Yao J., Zhang D. (2017). An investigation of obesity susceptibility genes in Northern Han Chinese by targeted resequencing. Medicine.

[22] Yu F., Zhu A.C., Liu S., Gao B., Wang Y., Khudaverdyan N., Yu C., Wu Q., Jiang Y., Song J. (2023). RBM33 is a unique m(6)A RNA-binding protein that regulates ALKBH5 demethylase activity and substrate selectivity. Mol. Cell.

[23] Zhang X., Yao Y., Han J., Yang Y., Chen Y., Tang Z., Gao F. (2020). Longitudinal epitranscriptome profiling reveals the crucial role of N(6)-methyladenosine methylation in porcine prenatal skeletal muscle development. J. Genet. Genomics.

[24] Shin M.C., Jung Y.H., Jeong Y., Oh A.R., Lee S.B., Kim K. (2023). Kctd17-mediated Chop degradation promotes adipogenic differentiation. Biochem. Biophys. Res. Commun..

[25] Skoblov M., Marakhonov A., Marakasova E., Guskova A., Chandhoke V., Birerdinc A., Baranova A. (2013). Protein partners of KCTD proteins provide insights about their functional roles in cell differentiation and vertebrate development. BioEssays.

[26] Rizk R., Devost D. (2024). KCTD Proteins Have Redundant Functions in Controlling Cellular Growth. Int. J. Mol. Sci..

[27] Bai M., Yin H., Zhao J., Li Y., Wu Y. (2019). miR-182-5p overexpression inhibits chondrogenesis by down-regulating PTHLH. Cell Biol. Int..

[28] Elli F.M., Mattinzoli D., Lucca C., Piu M., Maffini M.A., Costanza J., Fontana L., Santaniello C., Forino C., Milani D. (2022). Novel pathogenetic variants in PTHLH and TRPS1 genes causing syndromic brachydactyly. J. Bone Miner. Res..

[29] Scheffer-Rath M.E.A., Veenstra-Knol H.E., Boot A.M. (2023). A novel mutation in PTHLH in a family with a variable phenotype with brachydactyly, short stature, oligodontia and developmental delay. Bone Rep..

[30] Goldberg S.R., Georgiou J., Glogauer M., Grynpas M.D. (2012). A 3D scanning confocal imaging method measures pit volume and captures the role of Rac in osteoclast function. Bone.

[31] Gu Y., Williams D.A. (2002). RAC2 GTPase deficiency and myeloid cell dysfunction in human and mouse. J. Pediatr. Hematol. Oncol..

[32] Zhang X., Cai S., Chen L., Yuan R., Nie Y., Ding S., Fang Y., Zhu Q., Chen K., Wei H. (2019). Integrated miRNA-mRNA transcriptomic analysis reveals epigenetic-mediated embryonic muscle growth differences between Wuzhishan and Landrace pigs1. J. Anim. Sci..

[33] Serão N.V., Veroneze R., Ribeiro A.M., Verardo L.L., Braccini Neto J., Gasparino E., Campos C.F., Lopes P.S., Guimarães S.E. (2011). Candidate gene expression and intramuscular fat content in pigs. J. Anim. Breed. Genet..

[34] Alradi M., Askari H., Shaw M., Bhavsar J.D., Kingham B.F., Polson S.W., Fancher I.S. (2024). A long-term high-fat diet induces differential gene expression changes in spatially distinct adipose tissue of male mice. Physiol. Genom..

[35] Xia J., Qi X., Wu Y., Zhu B., Xu L., Zhang L., Gao X., Chen Y., Li J., Gao H. (2016). Genome-wide association study identifies loci and candidate genes for meat quality traits in Simmental beef cattle. Mamm. Genome.

[36] Zhang L., Liu J., Zhao F., Ren H., Xu L., Lu J., Zhang S., Zhang X., Wei C., Lu G. (2013). Genome-wide association studies for growth and meat production traits in sheep. PLoS ONE.

[37] Zhang J., Zhang Z., Liu X., Chai Y., Yang P., Li J., Huang Y., Li L., Huang W., Yang G. (2022). Copy number variation of WBP1L gene revealed its association with growth traits across Chinese cattle populations. J. Agr. Sci..

[38] Jiang J., Huang J., Gu J., Cai X., Zhao H., Lu H. (2019). Genomic analysis of a spinal muscular atrophy (SMA) discordant family identifies a novel mutation in TLL2, an activator of growth differentiation factor 8 (myostatin): A case report. Case Rep..

[39] Shen C., Zhou J., Wang X., Yu X.Y., Liang C., Liu B., Pan X., Zhao Q., Song J.L., Wang J. (2017). Angiotensin-II-induced muscle wasting is mediated by 25-Hydroxycholesterol via GSK3β signaling pathway. EBioMedicine.

[40] Wang R., Li Y., Lin Y., Chen D., Sheng X., Zhao N., Liu W. (2022). Cloning and expression characteristic analysis of goat ST13 gene. Sheng Wu Gong Cheng Xue Bao.

[41] Silva-Vignato B., Coutinho L.L., Cesar A.S.M., Poleti M.D., Regitano L.C.A., Balieiro J.C.C. (2017). Comparative muscle transcriptome associated with carcass traits of Nellore cattle. BMC Genom..

[42] Pan Y., Sun G., Li G., Chen S., Liu H., Li H., Mei C., Yang W., Zan L. (2025). Sex-specific microbiota associations with backfat thickness, eye muscle area, and rumen fermentation in Qinchuan cattle. BMC Microbiology.

[43] Tasdelen I., Berger R., Kalkhoven E. (2013). PPARγ regulates expression of carbohydrate sulfotransferase 11 (CHST11/C4ST1), a regulator of LPL cell surface binding. PLoS ONE.

[44] Lin C.W., Peng Y.J., Lin Y.Y. (2020). LRRK2 Regulates CPT1A to Promote β-Oxidation in HepG2 Cells. Molecules.

[45] Yu M., Arshad M., Wang W., Zhao D., Xu L., Zhou L. (2018). LRRK2 mediated Rab8a phosphorylation promotes lipid storage. Lipids Health Dis..

[46] Messling J.E., Agger K., Andersen K.L. (2022). Targeting RIOK2 ATPase activity leads to decreased protein synthesis and cell death in acute myeloid leukemia. Blood.

[47] Gao Z., Xu C., Fan H. (2022). Analysis of RIOK2 Functions in Mediating the Toxic Effects of Deoxynivalenol in Porcine Intestinal Epithelial Cells. Int. J. Mol. Sci..

[48] Hebbar P., Abubaker J.A., Abu-Farha M., Alsmadi O., Elkum N., Alkayal F., John S.E., Channanath A., Iqbal R., Pitkaniemi J. (2021). Genome-wide landscape establishes novel association signals for metabolic traits in the Arab population. Hum. Genet..

[49] Nagdy H., Mahmoud K.G.M., Kandiel M.M.M., Helmy N.A., Ibrahim S.S., Nawito M.F., Othman O.E. (2018). PCR-RFLP of bone morphogenetic protein 15 (BMP15/FecX) gene as a candidate for prolificacy in sheep. Int. J. Vet. Sci. Med..

[50] Paek H.J., Li Z.Y., Quan B.H., Yin X.J. (2023). Application of PCR-RFLP for quick identification of MSTN mutants in MSTN mutant pig breeding. Anim. Biotechnol..

[51] Parasar P., Bhushan B. (2023). Characterization of BoLA class II DQA and DQB by PCR-RFLP, cloning, and sequencing reveals sequence diversity in crossbred cattle. Anim. Biotechnol..

[52] Sundaramahalingam M.A., Amrutha C., Rajeshbanu J., Thirukumaran K., Manibalan S., Ashokkumar M., Sivashanmugam P. (2023). In silico approach for enhancing innate lipid content of *Yarrowia lipolytica*, by blocking the acyl-CoA oxidase-1 enzyme, using various analogous compounds of lipids. J. Biomol. Struct. Dyn..

[53] Zhang F., Xiong Q., Tao H., Liu Y., Zhang N., Li X.F., Suo X.J., Yang Q.P., Chen M.X. (2021). ACOX1, regulated by C/EBPα and miR-25-3p, promotes bovine preadipocyte adipogenesis. J. Mol. Endocrinol..

[54] Jiao Y., Zan L.S., Liu Y.F., Wang H.B. (2011). Molecular characterization, polymorphism of the ACOX1 gene and association with ultrasound traits in Bos taurus. Genet. Mol. Res..

[55] Lee J.K., Cho Y.M., Lee J.H. (2010). Association of Bovine CSRP3 and ACOX1 Genes with Carcass and Meat Quality Traits. Korean J. Agr. Sci..

[56] Dominguez-Castaño P., Marchi Maiorano A., Silva Lopes J.E., Vargas de Oliveira M.H., Michel Castilhos A., Vasconcelos Silva J.A.I. (2023). Genetic parameters for mouth size and their influence on growth traits in pasture-raised Nelore cattle. J. Anim. Sci..

[57] Chen S., Zhou Y., Chen Y., Gu J. (2018). fastp: An ultra-fast all-in-one FASTQ preprocessor. Bioinformatics.

[58] Li H., Durbin R. (2010). Fast and accurate long-read alignment with Burrows-Wheeler transform. Bioinformatics.

[59] Purcell S., Neale B., Todd-Brown K., Thomas L., Ferreira M.A., Bender D., Maller J., Sklar P., de Bakker P.I., Daly M.J. (2007). PLINK: A tool set for whole-genome association and population-based linkage analyses. Am. J. Hum. Genet..

