# Supplementary material for: Genome-Wide Association Studies of Growth and Carcass Traits in Charolais Cattle Based on High-Coverage Whole-Genome Resequencing
Source: Int J Mol Sci. 2025 Nov 25;26(23):11411. doi: 10.3390/ijms262311411 (PMC12692023; doi:10.3390/ijms262311411)
Supplement: Supplementary file 1 [file ijms-26-11411-s001.zip › ijms-3928380-supplementary.pdf]

Supplementary Materials

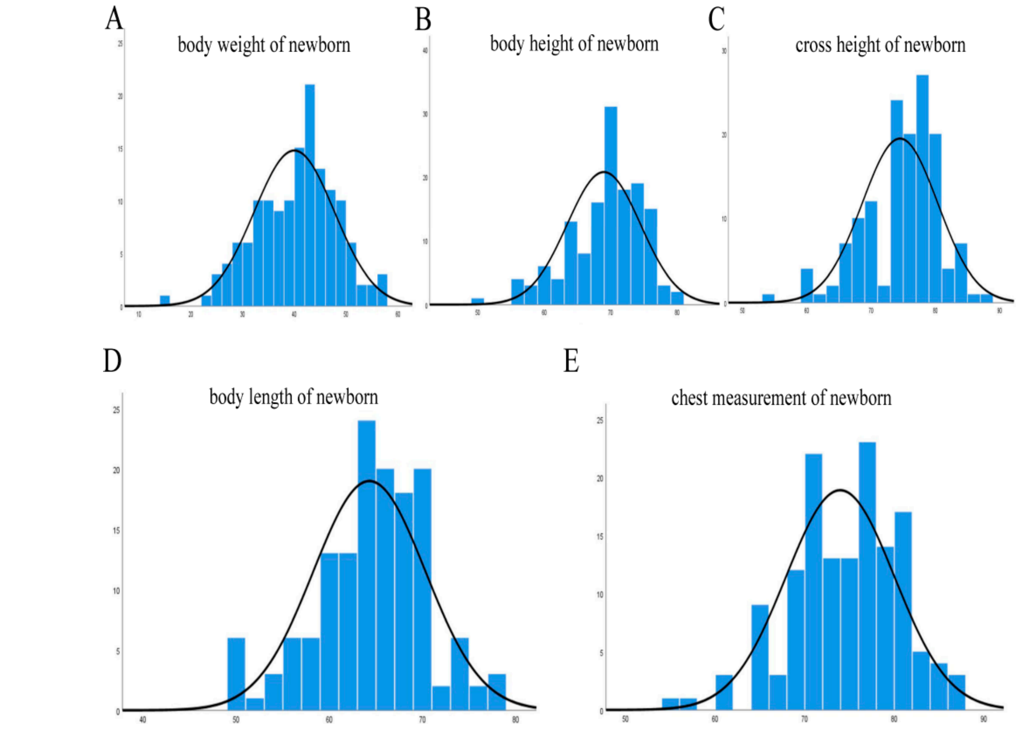

Figure S1. The phenotype normal distribution density of growth traits for newborn

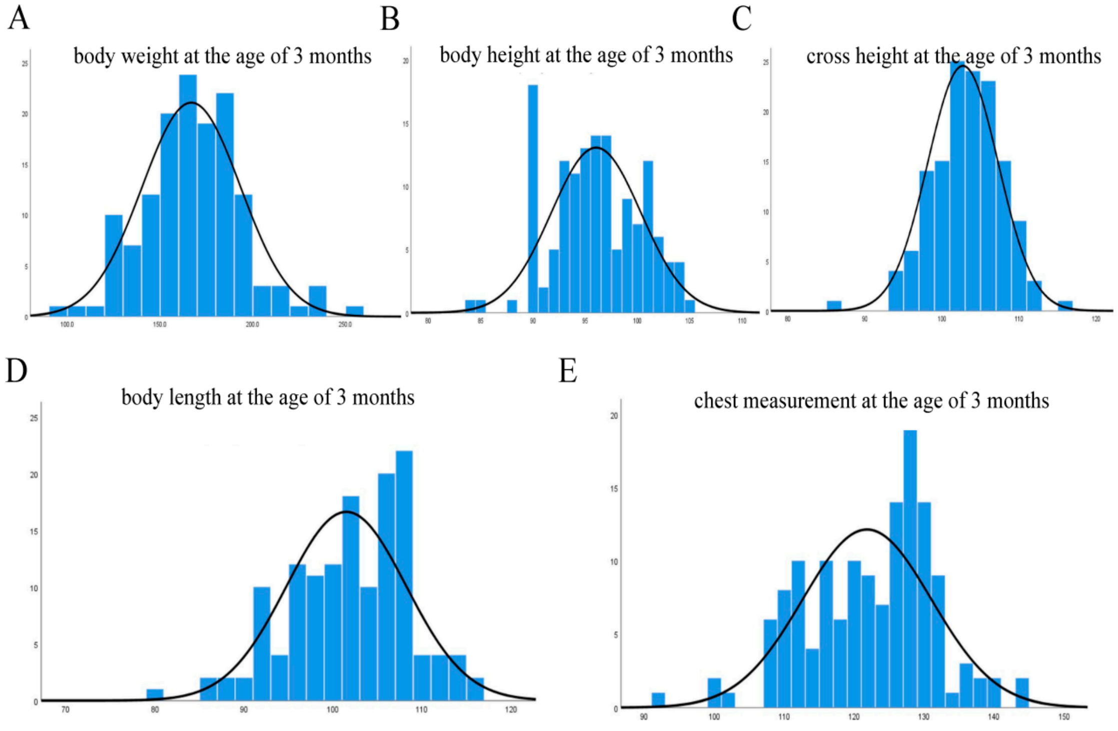

Figure S2. The phenotype normal distribution density of growth traits at 3 months of age

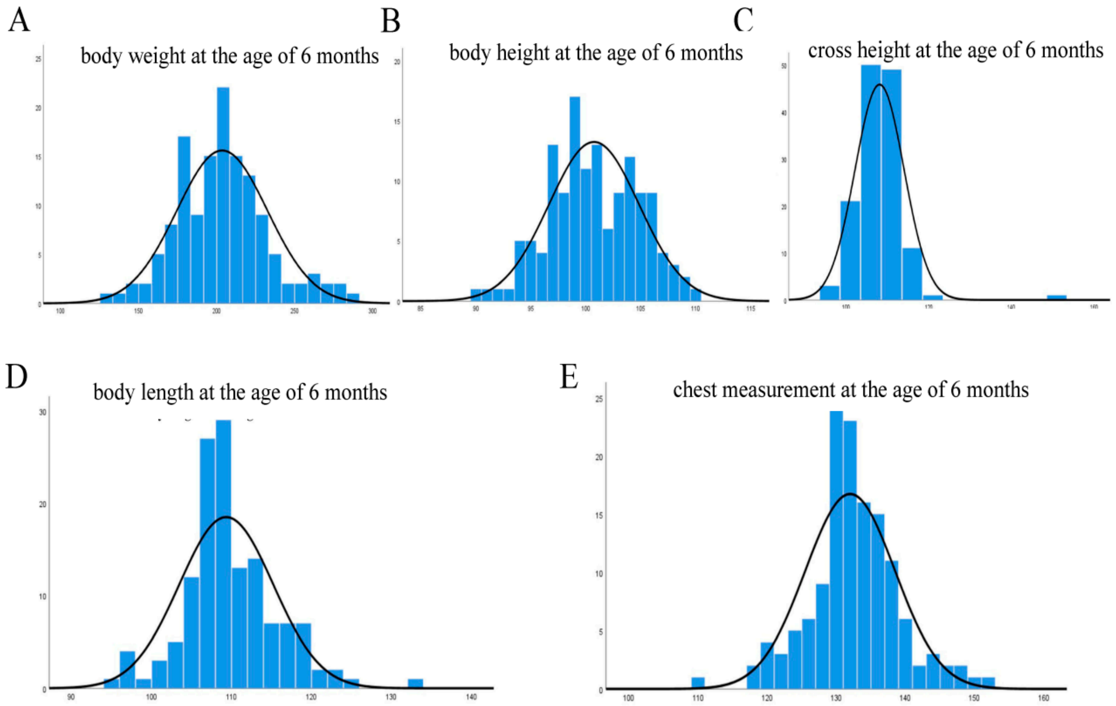

Figure S3. The phenotype normal distribution density of growth traits at 6 months of age

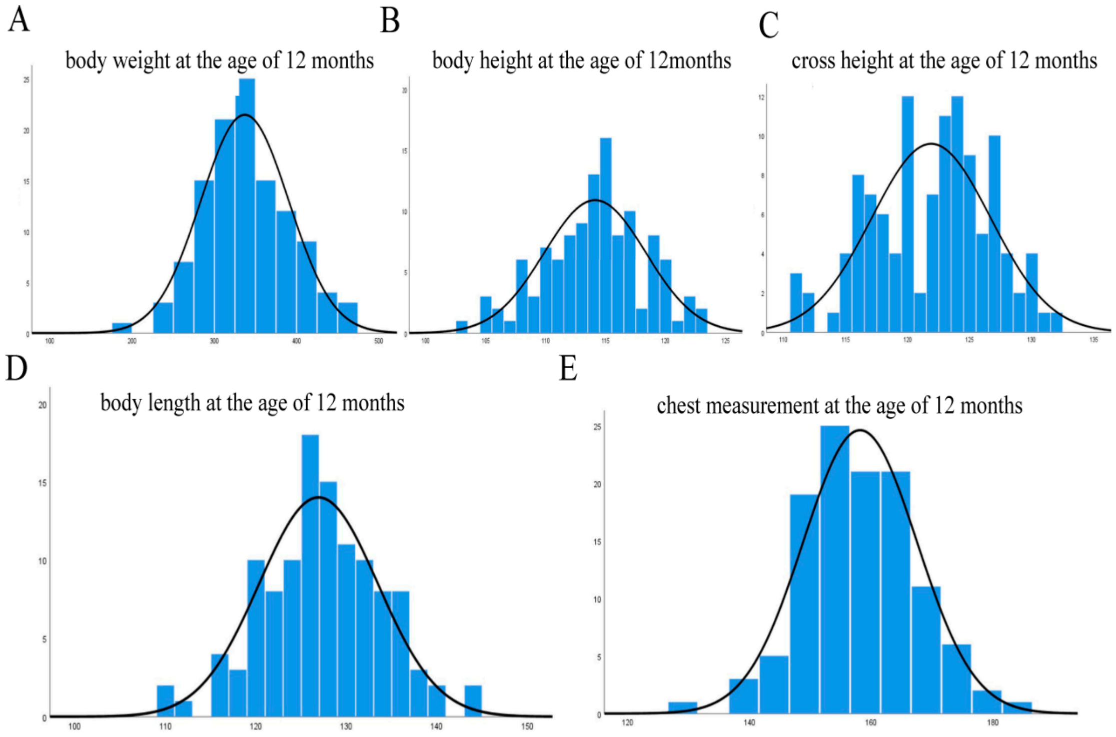

Figure S4. The phenotype normal distribution density of growth traits at 12 months of age

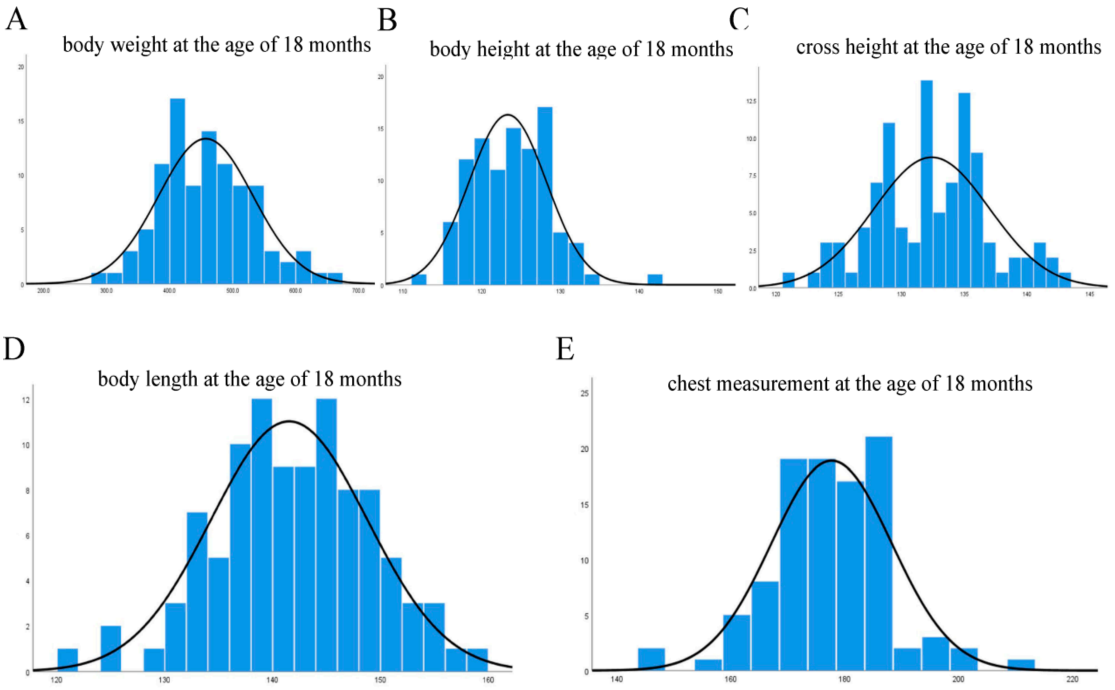

Figure S5. The phenotype normal distribution density of growth traits at 18 months of age

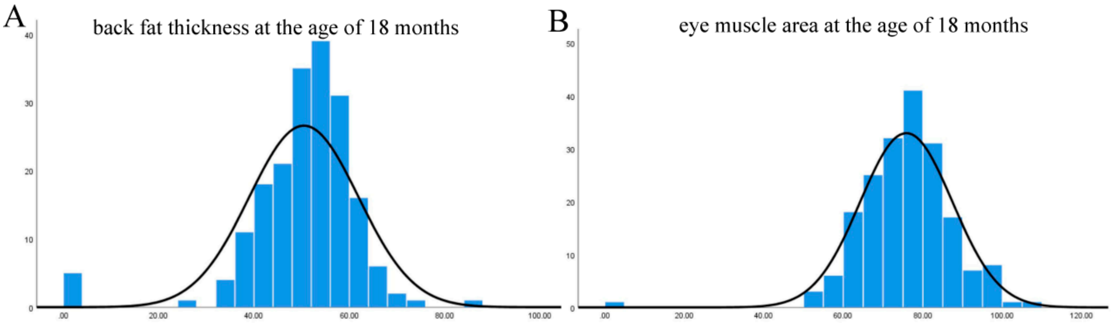

Figure S6. The phenotype normal distribution density of back fat thickness and eye muscle area at 18 months of age

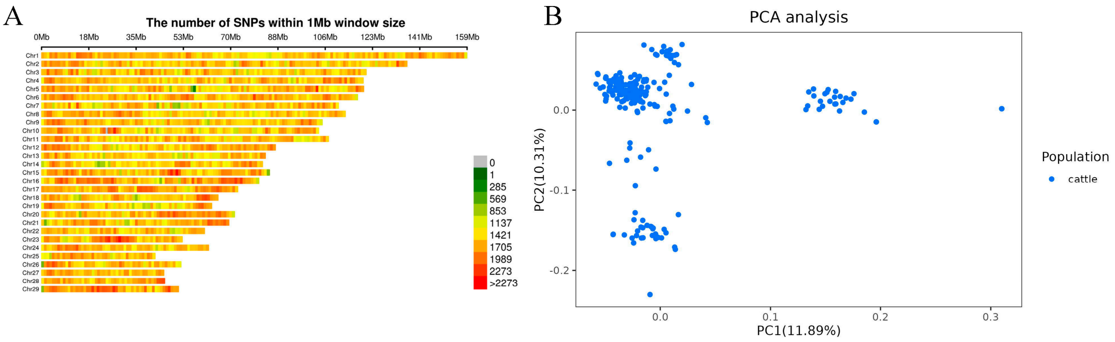

Figure S7. The SNP density and PCA of the Charolais cattle population. (A) The SNP density in the cattle genome within a 1Mb window size. (B) Plot of the first two principal components (PC1 and PC2) of the Charolais cattle population based on whole genome SNPs.

Table S1 Statistical analysis of phenotypic correlations for newborn

Traits	NB-BW(kg)	NB-BH(cm)	NB-CH (cm)	NB-BL (cm)	NB-CM (cm)
NB-BW (kg)	1	0.715^{**}	0.739^{**}	0.628^{**}	0.780^{**}
NB-BH (cm)	0.715^{**}	1	0.905^{**}	0.688^{**}	0.782^{**}
NB-CH (cm)	0.739^{**}	0.905^{**}	1	0.679^{**}	0.817^{**}
NB-BL (cm)	0.628^{**}	0.688^{**}	0.679^{**}	1	0.596^{**}
NB-CM (cm)	0.780^{**}	0.782^{**}	0.817^{**}	0.596^{**}	1

Note: NB-BW, body weight of newborn; NB-BH, body height of newborn; NB-CH, cross height of newborn; NB-BL, body length of newborn; NB-CM, chest measurement of newborn.

Table S2 Statistical analysis of phenotypic correlations for the age of 3 months

Traits	3-BW (kg)	3-BH (cm)	3-CH (cm)	3-BL (cm)	3-CM (cm)
3-BW (kg)	1	0.709^{**}	0.746^{**}	0.678^{**}	0.790^{**}
3-BH (cm)	0.709^{**}	1	0.804^{**}	0.726^{**}	0.718^{**}
3-CH (cm)	0.746^{**}	0.804^{**}	1	0.731^{**}	0.739^{**}
3-BL (cm)	0.678^{**}	0.726^{**}	0.731^{**}	1	0.826^{**}
3-CM (cm)	0.790^{**}	0.718^{**}	0.739^{**}	0.826^{**}	1

Note: 3-BW, body weight at the age of 3 months; 3-BH, body height at the age of 3 months n; 3-CH, cross height at the age of 3 months; 3-BL, body length at the age of 3 months; 3-CM, chest measurement at the age of 3 months.

Table S3 Statistical analysis of phenotypic correlations for the age of 6 months

Traits	6-BW (kg)	6-BH (cm)	6-CH (cm)	6-BL (cm)	6-CM (cm)
6-BW (kg)	1	0.717^{**}	0.544^{**}	0.770^{**}	0.833^{**}
6-BH(cm)	0.717^{**}	1	0.537^{**}	0.612^{**}	0.591^{**}
6-CH(cm)	0.544^{**}	0.537^{**}	1	0.479^{**}	0.508^{**}
6-BL(cm)	0.770^{**}	0.612^{**}	0.479^{**}	1	0.692^{**}
6-CM(cm)	0.833^{**}	0.592^{**}	0.508^{**}	0.692^{**}	1

Note: 6-BW, body weight at the age of 6 months; 6-BH, body height at the age of 6months n; 6-CH, cross height at the age of 6months; 6-BL, body length at the age of 6 months; 6-CM, chest measurement at the age of 6 months.

Table S4 Statistical analysis of phenotypic correlations for the age of 12 months

Traits	12-BW (kg)	12-BH (cm)	12-CH (cm)	12-BL (cm)	12-CM (cm)
12-BW (kg)	1	0.682**	0.760**	0.748**	0.858**
12-BH (cm)	0.682**	1	0.809**	0.597**	0.633**
12-CH (cm)	0.760**	0.890**	1	0.689**	0.692**
12-BL (cm)	0.748**	0.597**	0.689**	1	0.604**
12-CM (cm)	0.858**	0.633**	0.692**	0.604**	1

Note: 12-BW, body weight at the age of 12 months; 12-BH, body height at the age of 12 months n; 12-CH, cross height at the age of 12 months; 12-BL, body length at the age of 12 months; 12-CM, chest measurement at the age of 12 months.

Table S5 Statistical analysis of phenotypic correlations for the age of 18 months

Traits	18-BW (kg)	18-BH (cm)	18-CH (cm)	18-BL (cm)	18-CM (cm)
18-BW (kg)	1	0.554**	0.489**	0.623**	0.908**
18-BH (cm)	0.554**	1	0.727**	0.412**	0.532**
18-CH (cm)	0.489**	0.727**	1	0.396**	0.470**
18-BL (cm)	0.623**	0.412**	0.396**	1	0.593**
18-CM (cm)	0.908**	0.532**	0.470**	0.593**	1

Note: 18-BW, body weight at the age of 18 months;18-BH, body height at the age of 18 months n; 18-CH, cross height at the age of 18 months; 18-BL, body length at the age of 18 months; 18-CM, chest measurement at the age of 18 months.

Table S6 Significant SNP with putative candidate genes for growth and backfat thickness traits

**Traits**	**Chr**	**Location**	***P*-value**	**Genes**
NB-BW	4	6,570,100	1.22E-08	/
	4	117,307,166	2.98E-08	EN2, CNPY1, RBM33
	8	87,702,470	2.69E-09	/
	9	53,197,952	1.35E-09	FHL5, UFL1, ENSBTAG00000056105
	11	91,024,334	5.68E-09	SOX11
	12	6,436,206	6.02E-08	/
	14	4,477,205	3.67E-09	FAM135B
	15	64,714,146	7.20E-08	ENSBTAG00000059871, CAPRIN1, NAT10, ABTB2
	18	5,594,153	5.37E-08	WWOX

	19	9,802,072	6.66E-08	PPM1E, ENSBTAG00000046283
	21	14,191,734	1.15E-09	ENSBTAG00000048002, ENSBTAG00000054998, ENSBTAG00000022990/CHD2
	27	45,318,897	1.80E-08	/
NB-BH	4	84,072,197	1.55E-07	ENSBTAG00000006152, ENSBTAG00000059523
	9	12,333,503	2.04E-07	ENSBTAG00000059951, KCNQ5
	14	44,244,522	4.67E-08	PAG1
	15	8,436,636	4.13E-08	ARHGAP42
	15	8,475,863	1.11E-08	/
	22	4,195,711	7.68E-09	RBMS3
NB-CH	4	84,072,197	6.97E-08	ENSBTAG00000059523
	15	8,339,730	1.89E-07	ARHGAP42
	15	8,424,762	2.25E-07	ARHGAP42
	15	8,436,636	4.76E-08	ARHGAP42
	15	8,473,648	4.63E-08	/
	15	8,475,863	1.55E-08	/
NB-BL	2	12,775,835	2.50E-07	/
NB-CM	6	105,357,110	2.33E-07	WDR1
	9	12,298,541	5.94E-08	KCNQ5, ENSBTAG00000059951
	16	106,901	1.39E-07	/
	29	6,030,287	1.33E-08	FOLH1B, NOX4
3-BH	5	75,558,631	5.66E-08	KCTD17, TMPRSS6, IL2RB, SSTR3, C1QTNF6, RAC2
	5	76,987,691	1.55E-07	PKP2
	5	81,904,874	1.08E-07	PTHLH, ENSBTAG00000006530
	21	8,674,692	1.39E-07	ARRDC4
	23	20,548,719	1.38E-07	TNFRSF21, CD2AP
	23	20,746,524	2.51E-07	CD2AP, ADGRF2, ADGRF4. OPN5
	23	33,862,565	1.19E-07	/
3-CH	8	88,446,127	1.07E-07	GADD45G
	8	88,450,271	1.07E-07	GADD45G
	1	57,070,924	1.66E-07	SLC9C1, CD200
3-BL	1	110,115,447	1.76E-07	VEPH1, PTX3
	6	14,425,312	1.04E-07	/
	6	14,426,657	1.14E-07	/
	6	19,669,092	1.12E-07	ARHGEF38, ENSBTAG00000059903, PPA2
	26	9,852,663	1.10E-07	RNLS
	26	9,853,910	3.86E-08	RNLS
	26	9,854,220	1.10E-07	RNLS
	26	9,855,907	1.10E-07	RNLS
	26	10,946,345	2.04E-07	CH25H, LIPA, IFIT2, IFIT3

	26	17,752,324	1.79E-07	DNTT, OPALIN, TLL2
	26	17,752,911	4.13E-08	DNTT, OPALIN, TLL2
3-CM	1	136,442,772	1.36E-08	/
	1	143,624,511	1.83E-08	ENSBTAG00000057312, HSF2BP
	1	142,113,497	4.11E-08	RIPK4, PRDM15, C2CD2
	1	145,626,024	1.37E-07	PCBP3, COL6A1
	1	141,294,619	2.26E-07	ENSBTAG00000067615, BACE2, ENSBTAG00000066992
	3	120,853,447	2.10E-07	ING5, D2HGDH, GAL3ST2, NEU4, PDCD1, RTP5, FAM240C
	4	85,926,405	7.26E-08	WNT16, FAM3C
	4	88,017,825	2.11E-07	ASB15, LMOD2, WASL
	4	88,154,448	2.11E-07	WASL, ENSBTAG00000054421, HYAL4, SPAM1, ENSBTAG00000063333
	5	64,291,052	1.23E-07	SCYL2, SLC17A8, ENSBTAG00000062325, ENSBTAG00000052603, NR1H4
	9	4,005,854	4.19E-08	/
	9	97,488,537	5.62E-08	PRKN
	13	54,418,624	1.73E-07	BIRC7, YTHDF1, BHLHE23, SLC17A9, GID8, DIDO1
	13	54,456,313	1.83E-07	BHLHE23, SLC17A9, GID8, DIDO1, TCFL5, COL9A3
	16	77,698,012	1.74E-07	PTPRC, ENSBTAG00000046345
	19	27,027,195	2.74E-09	DLG4, ACADVL, DVL2, PHF23, GABARAP, CTDNEP1, ELP5, CLDN7, SLC2A4, YBX2. ENSBTAG00000058302, ENSBTAG00000059999, EIF5A, GPS2, NEURL4, ENSBTAG00000045892, ACAP1, KCTD11,TMEM95, TNK1, NLGN2, PLSCR3, TMEM256, SPEM2, SPEM1, TMEM102, FGF11,CHRNB1
	19	27,650,852	4.87E-09	CYB5D1, NAA38, CHD3, RNF227, KCNAB3, TRAPPC1, CNTROB, GUCY2D, ALOX15B, ALOX12B, ALOXE3
	19	27,067,495	5.97E-09	ELP5, CLDN7, SLC2A4, YBX2. ENSBTAG00000058302, ENSBTAG00000059999, EIF5A, GPS2, NEURL4, ENSBTAG00000045892, ACAP1, KCTD11,TMEM95, TNK1, NLGN2, PLSCR3, TMEM256, SPEM2, SPEM1, TMEM102, FGF11, CHRNB1, ENSBTAG00000050569, ZBTB4, POLR2A

19	27,029,767	7.70E-09	DLG4, ACADVL, DVL2, PHF23, GABARAP, CTDNEP1, ELP5, CLDN7, SLC2A4, YBX2. ENSBTAG00000058302, ENSBTAG00000059999, EIF5A, GPS2, NEURL4, ENSBTAG00000045892, ACAP1, KCTD11,TMEM95, TNK1, NLGN2, PLSCR3, TMEM256, SPEM2, SPEM1, TMEM102, FGF11,CHRNB1，ENSBTAG00000050569
19	27,104,179	8.16E-09	EIF5A, GPS2, NEURL4, ENSBTAG00000045892, ACAP1, KCTD11, TMEM95, TNK1, NLGN2, PLSCR3, TMEM256, SPEM2, SPEM1, TMEM102, FGF11, CHRNB1, ENSBTAG00000050569, ZBTB4, POLR2A, ENSBTAG00000066924
19	27,027,780	1.89E-08	DLG4, ACADVL, DVL2, PHF23, GABARAP, CTDNEP1, ELP5, CLDN7, SLC2A4, YBX2. ENSBTAG00000058302, ENSBTAG00000059999, EIF5A, GPS2, NEURL4, ENSBTAG00000045892, ACAP1, KCTD11,TMEM95, TNK1, NLGN2, PLSCR3, TMEM256, SPEM2, SPEM1, TMEM102, FGF11,CHRNB1，ENSBTAG00000050569
19	27,080,016	2.36E-08	SLC2A4, YBX2. ENSBTAG00000058302, ENSBTAG00000059999, EIF5A, GPS2, NEURL4, ENSBTAG00000045892, ACAP1, KCTD11, TMEM95, TNK1, NLGN2, PLSCR3, TMEM256, SPEM2, SPEM1, TMEM102, FGF11, CHRNB1, ENSBTAG00000050569, ZBTB4, POLR2A
19	27,105,388	2.36E-08	EIF5A, GPS2, NEURL4, ENSBTAG00000045892, ACAP1, KCTD11, TMEM95, TNK1, NLGN2, PLSCR3, TMEM256, SPEM2, SPEM1, TMEM102, FGF11, CHRNB1, ENSBTAG00000050569, ZBTB4, POLR2A, ENSBTAG00000066924
19	27,133,108	2.36E-08	ENSBTAG00000045892, ACAP1, KCTD11, TMEM95, TNK1, NLGN2, PLSCR3, TMEM256, SPEM2, SPEM1, TMEM102, FGF11, CHRNB1, ENSBTAG00000050569, ZBTB4, POLR2A, ENSBTAG00000066924, ENSBTAG00000053047

19	27,297,432	2.36E-08	ENSBTAG00000066924, ENSBTAG00000053047, TNFSF12, TNFSF13, SENP3, EIF4A1, CD68, MPDU1, SOX15, FXR2, SAT2, SHBG, ATP1B2, TP53, WRAP53
19	27,419,813	2.36E-08	SOX15, FXR2, SAT2, SHBG, ATP1B2, TP53, WRAP53, EFNB3, DNAH2
19	27,509,362	2.36E-08	DNAH2, ENSBTAG00000058660, KDM6B, TMEM88, CYB5D1, NAA38, CHD3, RNF227, KCNAB3
19	27,610,246	2.36E-08	DNAH2, ENSBTAG00000058660, KDM6B, TMEM88, CYB5D1, NAA38, CHD3, RNF227, KCNAB3, TRAPPC1, CNTROB, GUCY2D, ALOX15B
19	27,777,599	2.36E-08	ALOX15B, ALOX12B, ALOXE3, HES7, PER1, VAMP2, ENSBTAG00000059968, TMEM107, BORCS6, AURKB, CTC1
19	27,960,609	2.36E-08	CTC1, PFAS, ENSBTAG00000068110, RANGRF, ARHGEF15, ODF4, KRBA2, RPL26, RNF222, ENSBTAG00000051943, ENSBTAG00000048507, NDEL1
19	57,480,804	2.65E-08	/
19	27,051,734	3.14E-08	PHF23, GABARAP, CTDNEP1, ELP5, CLDN7, SLC2A4, YBX2. ENSBTAG00000058302, ENSBTAG00000059999, EIF5A, GPS2, NEURL4, ENSBTAG00000045892, ACAP1, KCTD11,TMEM95, TNK1, NLGN2, PLSCR3, TMEM256, SPEM2, SPEM1, TMEM102, FGF11,CHRNB1， ENSBTAG00000050569, ZBTB4
19	28,107,004	4.13E-08	ENSBTAG00000057943, NEDL1, MYH10
19	27,107,903	1.04E-07	EIF5A, GPS2, NEURL4, ENSBTAG00000045892, ACAP1, KCTD11, TMEM95, TNK1, NLGN2, PLSCR3, TMEM256, SPEM2, SPEM1, TMEM102, FGF11, CHRNB1, ENSBTAG00000050569, ZBTB4, POLR2A, ENSBTAG00000066924
19	27,442,602	1.95E-07	SAT2, SHBG, ATP1B2, TP53, WRAP53, EFNB3, DNAH2, ENSBTAG00000058660, KDM6B
21	27,288,507	1.86E-07	ENSBTAG00000068377
22	46,604,820	9.08E-08	CACNA2D3

	23	7,646,510	1.76E-07	KIFC1, PHF1, CUTA, SYNGAP1, ZBTB9, BAK1, GGNBP1
	23	7,643,460	2.30E-07	KIFC1, PHF1, CUTA, SYNGAP1, ZBTB9, BAK1, GGNBP1
	23	7,647,173	2.30E-07	KIFC1, PHF1, CUTA, SYNGAP1, ZBTB9, BAK1, GGNBP1
	24	47,500,024	1.97E-07	SMAD2
	27	16,929,407	1.92E-07	ENSBTAG00000066762
6-BW	16	67,296,178	8.00E-09	HMCN1, ENSBTAG00000054031
6-BH	1	40,469,475	6.81E-08	/
	1	40,470,433	5.94E-08	/
	1	40,489,259	2.64E-09	/
	1	46,808,309	6.14E-08	ZPLD1
	1	50,459,609	8.55E-08	CBLB
	1	55,722,481	1.83E-07	/
	3	109,859,706	2.34E-07	AGO3, AGO1, AGO4
	3	109,917,789	2.34E-07	AGO3, AGO1, AGO4
	3	110,110,382	2.34E-07	CLSPN, C3H1orf216, PSMB2, TFAP2E, NCDN, KIAA0319L
	3	110,127,355	2.34E-07	CLSPN, C3H1orf216, PSMB2, TFAP2E, NCDN, KIAA0319L, ENSBTAG00000058491
	3	110,145,138	2.34E-07	CLSPN, C3H1orf216, PSMB2, TFAP2E, NCDN, KIAA0319L, ENSBTAG00000058491
	3	110,149,401	2.34E-07	C3H1orf216, PSMB2, TFAP2E, NCDN, KIAA0319L, ENSBTAG00000058491
	3	110,172,532	2.34E-07	PSMB2, TFAP2E, NCDN, KIAA0319L, ENSBTAG00000058491
	3	110,206,022	2.34E-07	PSMB2, TFAP2E, NCDN, KIAA0319L, ENSBTAG00000058491
	3	110,220,049	4.70E-08	PSMB2, TFAP2E, NCDN, KIAA0319L, ENSBTAG00000058491
	3	110,247,996	2.34E-07	PSMB2, TFAP2E, NCDN, KIAA0319L, ENSBTAG00000058491, ZMYM4
	3	110,289,961	2.34E-07	TFAP2E, NCDN, KIAA0319L, ENSBTAG00000058491, ZMYM4
	5	28,154,221	1.85E-07	SCN8A
	5	95,248,408	9.59E-08	GUCY2C, PLBD1, ENSBTAG00000054625, H4C16
	5	95,269,950	4.70E-08	GUCY2C, PLBD1, ENSBTAG00000054625, ATF7IP
	6	105,167,706	5.44E-08	SLC2A9, ENSBTAG00000059992
	9	28,050,379	1.05E-07	TRDN

	10	5,228,721	7.06E-08	THOC3, CPLX2
	10	5,230,623	1.80E-07	THOC3, CPLX2
	10	10,140,275	2.35E-07	DMGDH, BHMT2, BHMT
	10	10,155,087	1.68E-07	DMGDH, BHMT2, BHMT
	10	10,219,181	1.77E-07	DMGDH, BHMT2, BHMT, JMY
	10	10,223,866	7.96E-09	DMGDH, BHMT2, BHMT, JMY
	10	73,611,718	2.32E-07	PRKCH
	13	69,837,905	2.05E-07	TOP1, PLCG1, ZHX3
	13	70,244,234	2.57E-08	CHD6
	13	70,260,156	1.32E-08	CHD6
	13	70,272,583	8.11E-09	CHD6
	13	70,273,759	1.32E-08	CHD6
	17	63,893,573	1.97E-07	MYO1H, FOXN4, ACACB, ALKBH2, ENSBTAG00000062277, ENSBTAG00000060357
	17	63,914,305	1.49E-07	MYO1H, FOXN4, ACACB, USP30, ALKBH2, ENSBTAG00000062277, ENSBTAG00000060357
	18	21,854,903	2.81E-08	CHD9, RBL2, AKTIP, RPGRIP1L
	19	28,899,723	3.63E-08	XTS8, CFAP52, USP43, DHRS7C, GLP2R
	20	45,183,318	1.02E-07	/
	22	25,658,400	2.20E-07	/
	22	25,672,772	2.20E-07	/
	24	51,825,014	7.85E-08	DCC
	25	24,919,736	1.50E-07	NSMCE1, IL4R, ENSBTAG00000070035, IL21R, GTF3C1
	29	4,286,680	2.20E-07	/
6-CH	5	112,181,330	1.63E-09	XPNPEP3, RBX1, ST13, SLC25A17, DNAJB7
	6	70,538,754	6.13E-09	KDR
	7	10,922,763	1.24E-21	ENSBTAG00000003528, ENSBTAG00000026148, ENSBTAG00000059944
	9	10,033,325	1.11E-11	B3GAT2, SMAP1, ENSBTAG00000062225
	10	93,106,855	3.01E-13	ENSBTAG00000056426
	12	5,398,443	1.90E-10	ENSBTAG00000063654
	19	60,153,356	1.90E-08	/
	27	43,588,550	9.52E-12	ZNF385D
6-BL	1	279,326	1.74E-12	ENSBTAG00000056266, ENSBTAG00000006648
	1	60,798,726	1.90E-13	LSAMP, ENSBTAG00000055835
	2	50,709,478	1.56E-13	/

	3	71,085,551	1.25E-07	/
	5	93,482,624	8.04E-08	ENSBTAG00000059104, MGST1
	9	8,234,039	2.37E-10	ENSBTAG00000069526
	13	8,210,066	9.57E-11	FLRT3, MACROD2
	17	4,423,622	1.69E-09	TRIM2, ENSBTAG00000060974
	20	53,637,972	1.13E-09	CDH12
	25	38,926,380	1.12E-10	FBXL18, TNRC18, SLC29A4, WIPI2
	26	23,457,280	1.68E-07	WBP1L, CYP17A1, BORC57, AS3MT
	28	45,651,995	1.02E-09	ZNF32, ENSBTAG00000070001, TMAM, ENSBTAG00000065725
6-CM	6	97,488,347	1.93E-09	TMEM150C, SCD5
	7	63,865,532	1.66E-09	/
	8	21,538,207	3.75E-08	/
	9	26,644,328	2.31E-10	ENSBTAG00000063517, NKAIN2
	10	26,008,299	6.70E-08	HNRNPC, OR5AU1, ZNF219, TMEM253, ARHGEF40, NDRG2, RNASE13, TPPP2, SLC39A2, ENSBTAG00000046482
	15	46,826,070	8.06E-10	CNGA4, FHIP18, CCKBR, OR56B42, OR56B42B, OR52B2, OR52W1, C15H11orf42
	28	25,729,579	1.62E-11	HKDC1, HK1, TACR2, TSPAN15
12-CH	4	26,509,947	1.68E-07	SNX13, PRPS1L1
12-BL	26	35,295,503	1.48E-07	ENSBTAG00000053538, FHIP2A, TRUB1
	26	35,296,164	1.83E-07	ENSBTAG00000053538, FHIP2A, TRUB1
18-CH	1	135,608,678	2.37E-07	RAB6B, SRPRB, TF, INHCA
	1	138,970,091	2.40E-07	ATP2C1
	20	24,824,816	2.78E-22	ARL15
18-BL	10	94,460,683	2.08E-07	/
	19	54,301,504	1.05E-07	/
	20	37,261,860	1.32E-07	NIPBL, ENSBTAG00000068518
	20	37,265,531	1.65E-07	NIPBL, ENSBTAG00000068518
	20	37,267,232	1.63E-07	NIPBL, ENSBTAG00000068518
	20	37,291,383	7.05E-08	NIPBL, ENSBTAG00000068518
	20	37,293,432	2.36E-07	NIPBL, ENSBTAG00000068518
	20	37,294,674	1.65E-07	NIPBL, ENSBTAG00000068518
	20	37,327,449	1.65E-07	NIPBL, ENSBTAG00000068518
	20	37,346,360	1.29E-07	NIPBL, ENSBTAG00000068518
	20	37,365,739	1.68E-07	NIPBL, ENSBTAG00000068518
	20	37,368,591	1.87E-07	NIPBL, ENSBTAG00000068518
18-CM	5	68,375,567	7.33E-08	CHST11, SLC41A2
	5	68,381,721	1.30E-07	CHST11, SLC41A2
	5	68,413,285	1.60E-07	SLC41A2
	9	59,877,259	1.80E-07	BACH2
	9	59,880,083	2.45E-07	BACH2

	9	59,881,180	1.58E-07	BACH2
	9	59,884,645	1.58E-07	BACH2
	9	59,887,198	1.59E-07	BACH2
	9	59,889,840	1.58E-07	BACH2
	9	59,919,853	7.52E-08	BACH2
	9	59,982,423	8.26E-08	BACH2
	9	60,021,862	2.25E-07	BACH2
	9	62,472,307	1.66E-07	RARS2, SLC35A1, CFAP206, C9H6orf163
	22	5,996,486	2.07E-07	STT38
18-BFT	5	35,591,562	1.02E-07	NELL2, ENSBTAG00000032150
	5	36,519,558	7.59E-08	TMEM117
	5	36,702,141	4.36E-08	TMEM117, TWF1, IRAK4, PUS7L
	5	36,751,543	1.02E-07	TWF1, IRAK4, PUS7L
	5	40,425,885	1.51E-07	ENSBTAG00000066266, ENSBTAG00000055810, LRRK2
	5	41,012,555	7.22E-09	SLC2A13
	5	41,015,233	1.06E-07	SLC2A13
	5	41,016,779	1.04E-07	SLC2A13
	5	41,809,052	2.16E-08	KIF21A
	5	61,224,636	5.35E-08	NEDD1, ENSBTAG00000051433
	5	68,155,316	8.97E-09	CHST11
	6	113,083,162	2.37E-07	ENSBTAG00000027825, ENSBTAG00000019584, ENSBTAG00000049123, MRFAP1L1
	6	113,086,926	7.02E-10	ENSBTAG00000027825, ENSBTAG00000019584, ENSBTAG00000049123, MRFAP1L1
	7	96,701,826	3.99E-08	RIOK2
	7	96,704,216	7.25E-09	RIOK2
	7	96,707,761	1.23E-08	RIOK2
	7	96,708,299	3.01E-09	RIOK2
	7	99,892,524	2.78E-08	ENSBTAG00000059942，ENSBTAG00000064337，ENSBTAG00000062296，FAM174A,ST8SAI4
	7	96,706,959	4.88E-08	PCSK1,CAST,ERAP1,ERAP2,LNPEP,RIOK2,LIX1,ENSBTAG00000003118,ENSBTAG00000057073
	8	68,433,228	1.14E-07	/
	8	68,594,156	3.24E-08	/
	10	306,089	4.86E-09	ENSBTAG00000017396
	10	311,325	4.86E-09	ENSBTAG00000017396
	10	311,625	4.86E-09	ENSBTAG00000017396
	10	312,364	5.08E-08	ENSBTAG00000017396

11	65,409,018	3.78E-08	/
11	65,425,563	1.56E-07	/
11	72,979,758	2.71E-08	CIB4, CIMIP2C, OTOF
27	37,847,965	1.28E-07	POMK, HGSNAT, INTS10
